# COVID-19 and science advice on the ‘Grand Stage’: the metadata and linguistic choices in a scientific advisory groups’ meeting minutes

**DOI:** 10.1057/s41599-022-01403-1

**Published:** 2022-12-24

**Authors:** Hannah Baker, Shauna Concannon, Matthias Meller, Katie Cohen, Alice Millington, Samuel Ward, Emily So

**Affiliations:** 1 University of Cambridge, Cambridge, UK; 2 The Technical University of Munich, Munich, Germany

**Keywords:** Language and linguistics, Science, technology and society

## Abstract

Science advice for governments attracted great scrutiny during the COVID-19 pandemic, with the public spotlight on institutions and individual experts—putting science advice on the ‘Grand Stage’. A review of the academic literature identified transparency, a plurality of expertise, the science and policy ‘boundary’, and consensus whilst addressing uncertainty as key themes. The Scientific Advisory Group for Emergencies (SAGE) has been the primary provider of coordinated scientific and technical advice to the UK Government during emergencies since 2009. Using the first 89 of SAGE’s meeting minutes (study period: 22 January 2020–13 May 2021), the ‘metadata’ and linguistic choices are analysed to identify how SAGE’s role and protocols are communicated. This includes understanding which experts were regularly taking part in discussions, the role of scientific experts in the science advisory system and their influence on policy choices, and the degree of consensus and uncertainty within this group of experts—all of which relate to the degree of transparency with the public. In addition, a temporal analysis examines how these practices, such as linguistically marking uncertainty, developed over the period studied. Linguistic markers indexing certainty and uncertainty increased, demonstrating a commitment to precise and accurate communication of the science, including ambiguities and the unknown. However, self-references to SAGE decreased over the period studied. The study highlights how linguistic analysis can be a useful approach for developing an understanding of science communication practices and scientific ambiguity. By considering how SAGE presents to those outside the process, the research calls attention to what remains ‘behind the scenes’ and consequently limits the public’s understanding of SAGE’s role in the COVID-19 response.

## Introduction

Science advice for governments attracted great scrutiny during the COVID-19 pandemic, with the public spotlight on institutions and individual experts (Farrar and Ahuja, 2021; Jasanoff et al., 2021; Landler and Castle, 2020; Pamuk, 2021; Pearce, 2020). The Scientific Advisory Group for Emergencies (SAGE) has been the primary provider of coordinated scientific and technical advice to the UK Government during emergencies since 2009 (Cabinet Office, 2012; Whitty and Collet-Fenson, 2021; Wilsdon, 2014). However, it had never convened over such a prolonged period (Haddon et al., 2020). At the same time, it has faced critical attention over its transparency, influence on unprecedented policy decisions such as implementing a national lockdown, and communicating uncertainty (Atkinson et al., 2020; Haddon et al., 2020; Horton, 2020; Jasanoff et al., 2021). Hence, our sole focus is on SAGE as the object of study in this paper.

For the first time during an ongoing emergency, the UK Government released SAGE’s advice in the form of meeting minutes into the public domain (Haddon et al., 2020; UK Government, 2020a). Our study uses SAGE’s meeting minutes to explore how they construct SAGE’s role and communicate SAGE’s protocols (study period: 22 January 2020–13 May 2021). A review of the academic literature identified transparency, a plurality of expertise, the science and policy ‘boundary’, and consensus whilst addressing uncertainty as key themes. This review included the search terms “scientific advisory groups” and/or “scientific expertise” and “COVID-19”; we also studied the Institute for Government (hereafter, abbreviated as IfG; Haddon et al., 2020) and the House of Commons Science and Technology Committee (abbreviated as STC; 2021) reports on the provision of UK scientific advice during the pandemic. By role, we consider SAGE’s function in the COVID-19 response i.e., as scientific advisers. For SAGE’s protocols, we consider the procedures their terms of reference advise them to follow (Cabinet Office, 2012), i.e., being transparent, including a plurality of experts, and ‘highlighting’ uncertainty and the degree of consensus. The four themes underpin the following research questions:

**RQ1**: Did SAGE’s approach towards transparency and plurality of expertise change throughout the study period?

**RQ2**: How is SAGE’s role constructed within their meeting minutes?

**RQ3:** How is consensus and uncertainty communicated within SAGE’s meeting minutes?

Our first research question considers whether SAGE’s claims of increased transparency (UK Government, 2020a) through shorter publication lags, and the existence of a ‘core group’ of experts are substantiated (see Haddon et al., 2020; STC, 2021). For this question, we investigate the ‘metadata’ of the meeting minutes. In our context, metadata refers to data providing information about the minutes, such as the minutes’ release date and the recorded attendance to the meetings. The second and third questions focus on the linguistic choices in SAGE’s minutes and the construction of authority, consensus, and uncertainty which can be derived from these choices. COVID-19 studies have already drawn on SAGE’s meeting minutes to analyse the coherence of policymaking and SAGE’s advice (Birch, 2021; Cairney, 2021; Evans, 2022; Haddon et al., 2020; STC, 2021). Our contribution departs from prior work by focusing on the use of the meeting minutes as a communicative device. We provide an analysis of the linguistic practices employed to communicate degrees of certainty, demonstrating how this and the publication practices may impact the public’s understanding of the science advisory system and scientific ambiguity.

Our empirical analysis indicates that first, although the advisory process has become more transparent, further steps could be taken, including a clearer picture of the specific expertise feeding into discussions. Second, although scientists advise and ministers decide (Atkinson et al., 2020; Clark, 2020), there is evidence of SAGE taking a stronger stance on particular issues and discussing policy choices. Third, although the number of voices feeding into discussions increased, SAGE’s minutes present a consensus view. Fourth, although the marking of uncertainty is encouraged in SAGE’s guidance (Cabinet Office, 2012), in the early stages of the pandemic explicit markers (and implicit) are limited in SAGE’s minutes in comparison to the latter stages, potentially due to SAGE becoming more confident about what was known and unknown.

We have structured the paper to first present the theoretical framing for our analysis: Hilgartner’s (2000) ‘Science on Stage’ metapahor and the key themes underpinning scientific advice to governments. We then discuss the linguistic practices which form a key part of our methodological thinking, including Hyland’s (2005) presentation of the ‘stance’ framework; followed by an overview of SAGE’s guidance and its relevance to the key themes. The methodology section further justifies our use of the meeting minutes as the data source and explains how we conducted the exploration. Our analysis aligns with the key themes before we present the discussion and conclusions.

## Scientific advice to governments

Science and Technology Studies (STS) literature has long established that the success of scientific advice is contingent on the credibility of the advice and the advisors (Hilgartner, 2000; Jasanoff, 1990). In taking up Erving Goffman’s (1959) dramaturgical metaphor, Hilgartner (2000) adapts the idea of performance to credibly fulfil a (social) role in the practice of an established and prestigious science advisory body. Central is the concept of ‘stage management’, describing the active control over which information (about the advice and its production) is accentuated on the ‘front stage’ and which is not visible to the audience on the ‘backstage’ (Hilgartner, 2000).

Applying the prism of Hilgartner’s (2000) ‘Science on Stage’ framework allows us to focus on the techniques used in providing the advice and how the public appearance of SAGE is shaped to underscore its role and authority. Other scholars have also used this framework. For example, Takahashi (2019) evaluates the improvised ways experts performed their authority while under significant public pressure during the Fukushima disaster. The front stage/backstage analogy helps discern which elements of the evolving science advisory process are made to be seen and which remain concealed, i.e., the level of transparency, one of our key themes. The analogy is relevant as transparency can play a critical role in developing (or undermining) public trust in the science advisory process (Ruiu, 2020). However, transparency has complications and limitations, including the potential harm to either companies or research participants if the information is confidential or sensitive, and its value-laden nature in policy, i.e., choices over the level of content (Elliott, 2021). Our other key themes also relate to the level of transparency. For example, scholars have encouraged transparency about experts’ disciplines within ongoing debates and advocacy for diversity, pluralism, and the co-production of knowledge in science advisory committees (Donovan, 2019; Elliott, 2021; Jasanoff, 2006; Mitchell, 2020; Moore and MacKenzie, 2020).

The relationships between politics, knowledge, science and governments are complex, with eminent challenges delineating boundaries between them (Boschele, 2020; Gieryn, 1983; Jasanoff, 2004, 1987; Pamuk, 2021). Moore and MacKenzie (2020) argue that whenever scientists provide advice to political leaders, they risk their authority as they are not in control of how others may use their advice to serve political ends—hence calls for a transparent process. Scientific advisory bodies are composed of independent scientists rather than elected politicians (Pamuk, 2021). This independence and detachment can be considered sources of scientific advisors’ authority and credibility (Owens, 2015). However, there are complications between neutrality (detachment from politics) and usefulness. If a scientific advisory committee tries to be more useful by offering judgements, it can compromise its neutrality (Pamuk, 2021). An alternative solution is to conceptualise scientific advisory committees as a site for deliberation, where the committee describes the implications of scientific findings e.g., offering policy choices—a role that Jasanoff (1990) and Owens (2015) cited by Pamuk (2021), suggest scientific advisory committees already perform in practice. Birch (2021) also references the concept of neutrality: ‘normative light advice’ is when committees do not recommend any specific policy options, whilst explicit policy recommendations constitute ‘normatively heavy advice’.

The concepts of uncertainty and consensus tie in with transparency, as scholars consider it to be ‘commonplace’ that scientists should be honest about uncertainty (John, 2018 citing Betz, 2013; Gelfert, 2013; Parker, 2014), whilst Pamuk (2021) considers the simplification of complex information for decision-makers and the public to be a crucial role of expert committees. Consensus carries great social weight as it signals the successful closure of disagreement and a shared judgement by the relevant scientific community (Jasanoff, 2015). For example, Hulme (2013) and Pearce et al. (2018) refer to the Intergovernmental Panel on Climate Change (IPCC) as a prime example of projecting a scientific consensus as authoritative. However, this consensus is tightly coupled with the trade-off to backstage productive disagreement and acknowledging uncertainty, at the dispense of meaningful discussion about political or policy alternatives. It is good practice in expert committees, such as the US Supreme Court or the Bank of England’s Monetary Policy Committee, to publish and explain differences in opinion among members (Pamuk, 2021; Stirling, 2010).

Scientific advisory groups’ communication of consensus and uncertainty elucidate their practices.

The main contribution of this paper is our empirical analysis of the metadata and linguistic choices in SAGE’s meeting minutes. Therefore, we now discuss linguistic practices and how these relate to both the communication of authority (relevant to the science-policy boundary theme) and uncertainty.

### Linguistic practices and the communication of authority and uncertainty

Several studies have investigated how certainty is linguistically expressed (e.g., Biber, 2006; Coates, 1987; Holmes, 1990; Rubin, 2006), observing that when “expressing an opinion, an evaluation or a subjective interpretation, lexical devices expressing degrees of certainty and conviction abound” (Holmes, 1982, p. 22). Various linguistic phenomena can “index the degree of certainty a person communicates about a given topic and the level of authority with which they deliver their message” (Myketiak et al., 2017, p. 142). For example, hedges downgrade authorial commitment, making a statement more tentative (e.g., epistemic modals such as “may” and adverbials such as “possibly”), while boosters or emphasisers (e.g., epistemic modals such as “will” and adverbials such as “obviously”) convey higher degrees of certainty. The modulation of certainty via hedges, boosters, and other linguistic markers has been extensively studied in spoken dialogue (Coates, 1987; Holmes, 1990), academic writing (Hyland, 2005, 2002), corporate communication (Hyland, 1998), news articles, and science writing (Kuhi and Rezaei, 2020; Poole et al., 2019; Shen and Tao, 2021).

Hyland’s (e.g., 2005, 2002) work on academic writing is of particular relevance, due to its concern with the ways writers “balance objective information, subjective evaluation and interpersonal negotiation”, and how these functions “in gaining acceptance for claims” (Hyland, 2005, p. 180). Hyland (2005) presents a framework that examines the specific linguistic features used in the presentation of ‘stance’, defined as:

“an attitudinal dimension [that] includes features which refer to the ways writers present themselves and convey their judgements, opinions, and commitments. It is the ways that writers intrude to stamp their personal authority onto their arguments or step back and disguise their involvement” (Hyland, 2005, p. 176).

Features incorporated in Hyland (2005) include self-references, attitude markers, boosters, and hedges. Attitude markers convey affective attitudes, such as agreement or importance, while self-references explicitly state the authorial position. Pronouns are the most common form of self-reference and have been explored in relation to authorial responsibility in the text (Hyland, 2002), medical discourse (Atkinson, 1999), and conversation (Heritage and Raymond, 2005; Lerner and Kitzinger, 2007). Hyland (2002) asserts that self-reference, typically achieved through personal pronouns and possessive adjectives, is associated with commitment and knowledge claims.

Concerning the COVID-19 pandemic specifically, Hyland and Jiang (2021) examine boosters and attitude markers in academic articles to explore the ‘hyping’ of academic scientific research (e.g., the use of promotional language to stress certainty or the significance of findings). Shen and Tao (2021) use Hyland’s (2005) stance framework to conduct a corpus-based comparative study of medical and newspaper articles about the pandemic. They found stance markers were more common in the newspaper genre, but both genres employed tentative stance markers such as hedges to qualify or constrain their claims due to the lack of available evidence or information.

We argue that the minutes from SAGE meetings are critical historical documents providing a record of SAGE’s evolving ‘front stage’ performance. Other such records may be the advisors’ attendance at public briefings, interactions with traditional and social media, or (interview) contributions to institutional reports and STC evidence sessions. To continue a thought from Obermeister (2020), the scientific advisors not only had to learn how to advise and influence policymakers in the specific situation of a public health crisis (COVID-19) but also to negotiate their role while increasingly being in the public spotlight. We thus investigate the metadata and linguistic choices during a period in which SAGE’s minutes switched from the ‘backstage’ to the ‘front stage’. To contextualise this, the next section provides an overview of SAGE’s guiding documents (which are available in the public domain) and their relevance to the four key themes.

### SAGE on the ‘Grand Stage’

The UK is a pioneer in the way countries now identify and prepare for risks, as it was one of the first nations to appoint a Chief Scientific Advisor (CSA) and Chief Medical Officer (CMO) (Doubleday and Wilsdon, 2012; Haddon et al., 2020; Wilsdon, 2014;). SAGE exists within an extensive system of advice to the UK government (Wilsdon, 2014). For example, The Civil Contingencies Committee (COBR) is responsible for coordinating central Government decision-making during emergencies and for the activation of SAGE (Haddon, 2020; STC, 2021). The first SAGE meeting about the novel coronavirus occurred on 22 January 2020. The first COBR meeting was held on 24 January 2020 (Haddon and Ittoo, 2020). The World Health Organisation (2020a) announced COVID-19 as the name of this new disease on 11 February 2020 and declared it a global pandemic on 11 March 2020 (World Health Organisation, 2020b).

**Fig. 1:**
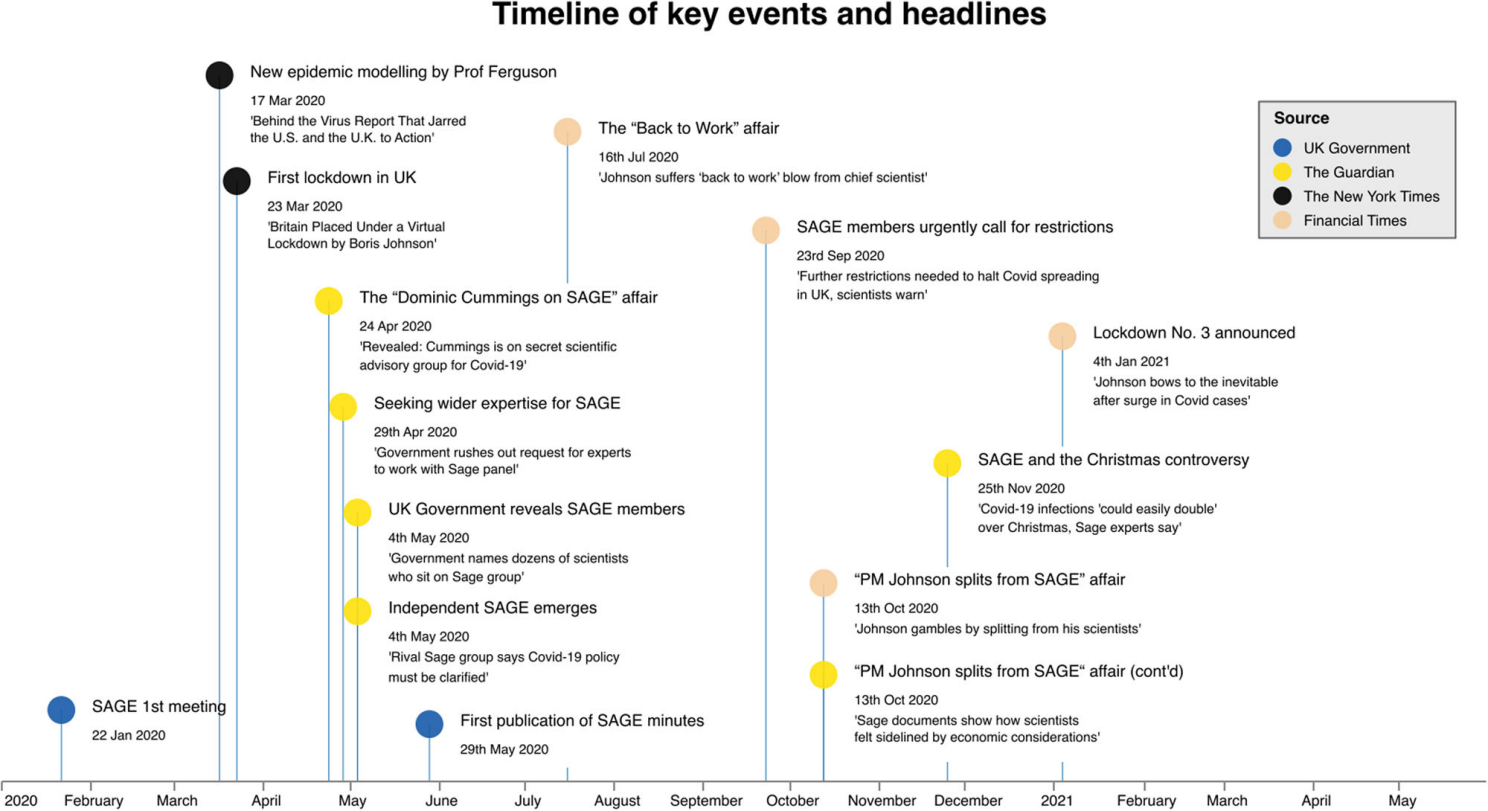
Timeline of key events and headlines identified, based on a media review of The Guardian, The New York Times, and the Financial Times between January 2020 and May 2021. Titles of articles as quotes, and dates denote the publication of the article. See Supplementary Information 2 for reference data and the corpus of reviewed media articles.

Numerous headlines focusing on SAGE, or the individual scientific advisors (see Fig. 1) suggest an increased awareness by the media of the scientific committee. According to Google (2022) Trends data (see Supplementary Information 1), relative interest in searching for SAGE in the UK, rose significantly in the week before the first lockdown and reached an absolute peak between mid-April and early May 2020. The interest later steadied to see further peaks in October 2020 and the first week of January 2021^1^. By this token, we contend that the UK’s science advisory system has hardly been more in the public spotlight—in other words, on the ‘Grand Stage’—ever before.

The ‘Enhanced SAGE guidance’ (Cabinet Office, 2012; hereafter abbreviated as ‘the Guidance’), which is currently available under the heading ‘Terms of Reference’ in the ‘About Us’ section on SAGE’s website (UK Government, 2021a) is referred to as a guiding document for SAGE in the Code of Practice for Scientific Advisory Committees and Councils (CoPSAC) (Government Office for Science, 2021)^2^. Within this, each of our key themes is discussed, which we now analyse in turn.

The Guidance refers to transparency as an “important element of democratic decision making” and states that “the evidence used to inform decisions should be published” (Cabinet Office, 2012, p. 23). In previous SAGE events, the public did not have access to the minutes until the emergency ceased (STC, 2021). Reports reflecting on SAGE’s involvement in previous crisis events have advocated for greater transparency, with Donovan (2021) noting that transparency dominated the STC (2011) report on the Volcanic Ash Crisis and Swine Flu. Methods to increase SAGE’s transparency included a greater openness about the selection of experts and releasing the minutes (Donovan, 2021; STC, 2011). After initial appeals to release the names of the experts during the COVID-19 pandemic, a letter from Sir Patrick Vallance (2020), the Government’s CSA, outlined the decision not to disclose SAGE membership. He cited reasons to safeguard individuals from lobbying and unwanted influence. However, on 4 May 2020, the Government disclosed the names, after giving advisors the option to opt-out. The Government released the minutes on 29 May 2020, accompanied by a press release, citing the unique situation of the pandemic as justification:

“Given the exceptional nature of the COVID-19 pandemic, the government, SAGE and its participants want to ensure there is as much available evidence and material as possible to the general public so there is full transparency on how science advice is being formulated.” (UK Government, 2020a).

A brief explainer of SAGE published during the pandemic (also available in the ‘About Us’ section on SAGE’s website) notes that “expert participation changes for each meeting, based on the expertise needed to address the crisis the country is faced with” (UK Government, 2020b). However, several scholars have criticised insufficiently diverse expertise and the reliance on a small group of experts during COVID-19 (Donovan, 2021; Moore and MacKenzie, 2020; Koppl, 2021). The IfG’s interviewees said there were “too few ‘dissenting voices” (Haddon et al., 2020, p. 33) and their report claims that, despite large numbers of people attending SAGE meetings or listed on the SAGE website, a ‘core group’ of medical scientists and modellers largely dominated discussions (Haddon et al., 2020). The STC (2021) report also refers to the concept of a ‘core group’.

According to the Guidance (Cabinet Office, 2012), SAGE advice is likely to be sought to weigh up the scientific and/or technical arguments and implications for/against policy options defined by others than the science advisers. This ‘boundary’ between science and policy is also evident in the CoPSAC (Government Office for Science, 2011, p. 32; 2021): “Scientific advisers should respect the democratic mandate of the Government to take decisions based on a wide range of factors and recognise that science is only part of the evidence that Government must consider in developing policy”. Witnesses for the public evidence sessions of the STC (2021) conveyed that SAGE participants understood the separation between distiling up-to-date scientific evidence and directing policy decisions (Clark, 2020). However, Boschele (2021, p.1) states COVID-19 has highlighted the “long-standing tensions between technocracy and democracy”. One of the IfG’s criticisms of the decision-making process was that it is unclear how scientific advice translated into political decisions in practice (Haddon et al., 2020).

The Guidance (Cabinet Office, 2012) also states that SAGE’s advice is likely to be sought on the degree of consensus and differences in opinions, i.e., if all or few experts agree; and the degree and cause of uncertainty (with confidence intervals and margins of error given as examples). SAGE is a committee, and meeting attendees will likely have varying views on topics. The aim of the SAGE committee, according to the Guidance, is to “ensure that coordinated, timely scientific and/or technical advice is made available to decision-makers to support UK cross-government decisions in COBR” (Cabinet Office, 2012, p. 12). During the COVID-19 pandemic, the STC (2021) report found that SAGE operated by providing a ‘central view’. Haddon et al. (2020) found that SAGE’s minutes rarely recorded internal disagreements. However, Professor Chris Whitty, England’s CMO, has highlighted the salience of an average scenario in reference to the communication of statistical confidence and uncertainty: “One of the things you are trying to convey is both the central projection—this is where on average we think things will go—and the uncertainty around that.” (STC, 2021, p. 22). Whitty told the STC (2021) that if there are dissenting opinions or a range of quantitative uncertainty, these opinions are conveyed to policymakers in a comprehensible way, whilst Vallance stated that SAGE tries to be transparent and open about how a consensus view is formed.

The SAGE secretariat is responsible for recording the minutes, which should follow standard practice for a science advisory committee (Cabinet Office, 2012), SAGE members then approve these for technical accuracy before publication. Both CoPSACs from 2011 and 2021 (Government Office for Science, 2011, 2021) state that minutes should accurately reflect proceedings, and generally be written in an unattributable form and in a way that is easy for members of the public to understand. Sensitive information can be placed in an undisclosed annexe. They additionally state differences in opinion and interpretation should be recorded in an impartial form in the minutes. Beyond this, and the statements about ‘highlighting’ disagreement (p. 47) and uncertainty (p. 33), we did not identify any guidance on linguistic practices for the meeting minutes within the Guidance for SAGE (Cabinet Office, 2012).

Now that we have summarised existing theoretical literature and SAGE’s guidance related to the key themes underpinning our research questions, we move to our methodology.

## Methodological approach

To understand SAGE’s ‘front stage’ performance, we use publicly available data: SAGE’s online meeting minutes (UK Government, 2021b)^3^ from the first meeting on 22 January 2020 to the 89th on 13 May 2021. We have focused on SAGE as they were the primary provider of scientific knowledge during the COVID-19 pandemic (UK Government, 2020b; Whitty and Collet-Fenson, 2021). The rationale for our study period is that it covered a cycle of unknowing (when the virus first emerged) to one of increased resolution when the UK was easing lockdown measures after the 2020–21 winter months and the vaccination programme was underway. The following sections motivate the use of the minutes as our data source and the mixed-methods analysis.

### Data source: SAGE’s meeting minutes

Meeting minutes are a particular format of writing: they convey the record and outcome of a meeting, yet at the same time they are more than a ‘mere’ transcript. The English Dictionary offers two definitions that highlight the dual nature of minutes: (a) *“*a summarised record of the proceedings at a meeting”, and (b) “an official memorandum authorising or recommending a course of action” (Lexico, 2021). Accordingly, meeting minutes provide an institutionally approved account that includes evaluative judgements on situations and normative assessments of possible actions.

Academic debates, including those on central banking and monetary policy, recognise that meeting minutes are a key form of communication and transparency (El-Shagi and Jung, 2015; Jung, 2016; Reeves and Sawicki, 2007; Sack and Kohn, 2003). We posit that SAGE’s minutes were also an essential form of transparency and communication during the COVID-19 crisis. Additionally, the press release for their initial release stated that Ministers receive advice from SAGE in the form of these minutes, alongside verbal contributions from the Government’s CSA and CMO in COBR and other Ministerial meetings (UK Government, 2020a).

COVID-19 studies have already begun to draw on meeting minutes as a source of information for analysis (Birch, 2021; Cairney, 2021; Haddon et al., 2020; STC, 2021; Shimizu and Negita, 2020). Birch (2021) uses examples within SAGE’s minutes when distinguishing between ‘normatively light advice’ and ‘normatively heavy advice’, and Cairney (2021) used SAGE’s meeting minutes, alongside papers and oral evidence, to create a narrative of the first UK lockdown, including changes in the level of caution expressed. Our analysis employs two methods using SAGE’s minutes as the primary data source to answer our central research questions.

### Method 1: SAGE’s approach towards transparency and plurality of expertise

We analysed the minutes’ metadata to answer our first research question, ‘Did SAGE’s approach towards transparency and plurality of expertise change throughout the study period?’. We thereby extended the analysis of the IfG (Haddon et al., 2020) and STC (2021) reports quantitatively. We were interested in finding continuations of delays to publications of meeting minutes and verifying the existence of a ‘core group’ of experts attending SAGE meetings.

We recorded the meeting and publication dates and created an ‘expert database’ documenting the meeting attendees. We identified their primary institutional affiliation and if each participant was a ‘scientific expert’, ‘observer’, or part of the secretariat—the roles listed and explained in an addendum to SAGE’s minutes. We then identified the number of meetings attended by each of the attendees listed within the minutes^4^.

Table 1 defines the series of timeframes chosen to reflect key moments over time i.e. when SAGE received a notable increase in attention through traditional broadcasting and publishing outlets. We chose these key moments under the assumption that external events were likely to change the self-reflection and positionality of SAGE, with implications for the drafting of the minutes. Substantiated by a media preview of *The Guardian*, the *Financial Times*, and *The New York Times* (shown previously in Fig. 1), which analysed articles about or directly referring to SAGE (i.e., more than only in a minor paragraph), the turning points identified were: the public release of Professor Ferguson’s Imperial epidemic modelling (16 March 2020), the first official publication of SAGE’s minutes (29 May 2020), the first definite advice for a ‘circuit-breaker’ lockdown (21 September 2020), and the run-up to the third lockdown in early January 2021 (22 December 2020). We used these four pivotal moments to segment our corpus of minutes into the five subgroups for temporal analysis.

**Table 1:** Timeframes used for analysis.

Timeframes	Meeting numbers	Meeting dates	Key moment likely to change the positionality of SAGE (applicable to *latter* date)
TF1	1–16	22 January 2020–16 March 2020	Publication of Professor Ferguson’s Imperial epidemic modelling.
TF2	17–39	18 March 2020–28 May 2020	Launch of SAGE’s minutes’ publication scheme on 29 May 2020.
TF3	40–58	4 June 2020– 1 September 2020	SAGE strongly advising “circuit-breaker” lockdown.
TF4	59–74	24 September 2020–22 December 2020	Run-up meeting to third lockdown (announced 4 January 2021).
TF5	75–89	7 January 2021– 3 May 2021	End of our study period.

Timeframes reflect key moments when traditional broadcasting and publishing outlets made scrutiny of SAGE apparent (see Fig. 1).

### Method 2: SAGE’s self-representation and approaches towards uncertainty

To explore the second and third research questions ‘How is SAGE’s role constructed within their meeting minutes?’ and ‘How are consensus and uncertainty communicated within SAGE’s meeting minutes?’, our research seeks insight into the linguistic choices made within the minutes. Specifically, we focus on linguistic markers that reflect self-presentation and uncertainty. Informed by Hyland’s (2005) framework, we examine how the meeting minutes mark the authorial position of SAGE and expressions of certainty. We study SAGE’s self-references to investigate how they portray SAGE’s role in the COVID-19 response. There were no instances of first-person singular pronouns in our sample. Plural forms (i.e., we, us, our) were also extremely rare (only ‘we’, (*n* = 16)). Additionally, ‘we’ refers both to SAGE (e.g., “we do not have reliable data”) and UK society more generally (e.g., “we may be further ahead on the epidemic curve”). However, we observe the explicit marking of self-reference to SAGE as in “SAGE advised…”. Consequently, we include only explicit self-reference to SAGE for the analysis, excluding all references to SAGE beyond the self-reference context (e.g., “X to respond to SAGE comments”, “ahead of discussion at SAGE next week”)^5^. In total, we identified 764 self-references of SAGE. Using the self-references, we conducted an inductive analysis to identify examples of SAGE’s portrayal of their role within the COVID-19 response—either implicitly or explicitly. Additionally, we compared the keywords (available in Supplementary Information 3) that characterise the SAGE self-representation sentences in each timeframe^6^.

We built on Shen and Tao (2021) to generate an initial list of stance markers and manually inspected the use of each marker^7^. We also assessed whether each marker performed hedging, boosting or attitudinal function in the context of our data, and added any additional markers specific to our corpus—this includes the explicit mention of uncertainty through confidence intervals labels (e.g., “low confidence”) introduced in the data partway through our study period. For markers observed to perform multiple functions (e.g., epistemic modals such as “will” that can be used as a booster or to talk about possible future scenarios) two researchers checked and classified each instance. The final counts only include those instances in which both annotators agreed functioned exclusively as either a hedging, booster, or attitude marker^8^. The complete list of markers used is available in Supplementary Information 4.

Frequencies for each marker were computed and normalised according to a standard text length of 1000 words (i.e., the raw frequency of each marker was divided by the total word count and multiplied by 1000). To ascertain whether the marking of certainty changed over time, we counted the frequencies and compared them across our proposed timeframes. Descriptive statistics are used to analyse the variance across the timeframe groupings. As the data is not normally distributed, statistical analysis using the non-parametric, independent samples Kruskal–Wallis tests (Kruskal and Wallis, 1952) are applied to analyse variance using the SPSS statistics package (IBM Corp., 2020). The Kruskal–Wallis test compares several groups (timeframes) in terms of a quantitative variable (linguistic markers). It tests the probability that a random observation from each group is equally likely to be above or below an equivalent observation from another group by comparing mean ranks. Where the non-parametric independent samples Kruskal–Wallis test observe a significant variation across groups, post hoc multiple comparison testing using Bonferroni Correction is applied for pairwise comparisons between individual timeframes. Other linguistic studies of corpora, e.g., Birhan (2021), use similar applications of statistical analysis.

### Ethical approval and informed consent statement

This article does not contain any studies with human participants performed by any of the authors. Therefore, informed consent was not deemed necessary. All data analysed is publicly available under an Open Government Licence v.3.0, and SAGE’s meeting minutes are in themselves unattributable. Although SAGE’s website and minutes provide the names of experts and their institutions as attendees, we have anonymised people’s names in our analysis. The analysis identifies general trends, not the content of specific individuals. We, therefore, do not anticipate that the data collected and analysed carries a likelihood of substantial damage or distress to these individuals from the data processing. In our literature review and discussion sections, we have only included the names of individuals with significant media presence during the pandemic and provided relevant references to secondary sources. We have stored data on either password-protected computers or a secure cloud-server. For these reasons, the lead author’s host institution, the Centre for Research in the Arts Social Sciences and Humanities (CRASSH), University of Cambridge, approved the process followed and did not require further formal ethical approval from a higher level within the University.

## Analysis of SAGE’s meeting minutes

In the following sections, we provide evidence, i.e., excerpts from the minutes exemplary for our various aspects of analysis. All emphasis, i.e., bold text and acronyms expanded as square brackets, in the quotations is ours to help emphasise and explain the points made.

### SAGE’s approach towards transparency

**Fig. 2:**
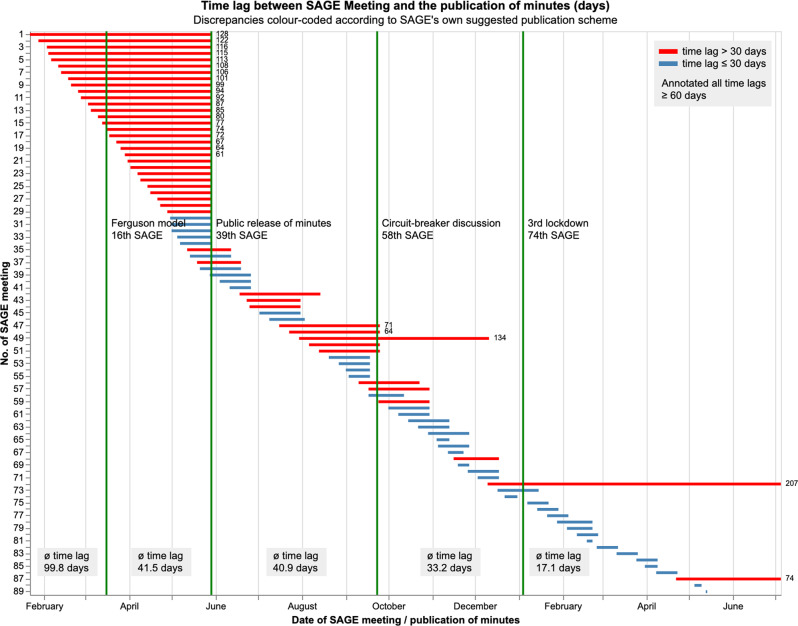
Discrepancy between the date of SAGE meetings and publication of meeting minutes according to gov.uk. Red indicates meeting minutes published 30 days after the meeting. Diagram inspired by the figure in Haddon et al. (2020, p. 42). This updated version uses meeting and publication data provided in Supplementary Information 5. Original data source: SAGE Meeting Minutes (1–89) from UK Government (2021b).

The Government’s public release of SAGE’s experts’ names and their meeting minutes increased SAGE’s transparency. Figure 2 extends the analysis presented in the IfG report (Haddon et al., 2020, p. 42) to the minutes contained within our study period. It shows the discrepancy between the date of SAGE meetings and the publication of meeting minutes according to gov.uk. We can confirm the delay in their release, which Haddon et al. (2020) noted. Our analysis found that the average delay for release in each of our timeframes (TF) decreased. Mean values being: TF1—99.8 days; TF2—41.5 days; TF3—40.9 days; TF4—33.2 days; and TF5—17.1 days. The latter two were within the 30-day target specified by SAGE (2020). Nonetheless, there are several outliers (shown in red), with some release delays greatly exceeding 30 days. An additional observation is that the minutes became available in HTML format beyond solely PDF during our study period, increasing accessibility and the possibility of analysis.

Our qualitative analysis of SAGE’s self-references (see the section “Method 2: SAGE’s self-representation and approaches towards uncertainty”) also indicates SAGE’s position on transparency. This included discussions about the release of Ferguson’s modelling (Meeting 15—13 March 2020) and endorsing the release of participants’ names (Meeting 29—28 April 2020). We also identified examples further supporting the release of information later in the pandemic:

“SAGE highlighted the importance of getting the maximum amount of information into the public domain for people to understand the epidemic in totality.” (Meeting 45—2 July 2020)

Throughout the study period, SAGE has demonstrated a more transparent approach by releasing experts’ names and meeting minutes. We have identified examples of SAGE’s position on transparency. However, it is not evident from the minutes alone who makes the final decision on the content and time of information releases, e.g., is this SAGE as an independent advisory body or informed by COBR and/or other Government bodies? One further step towards transparency would be including an accurate representation of the scientific expertise feeding into the meetings on SAGE’s website, which we now discuss.

### ‘Core group’ and plurality of voices in SAGE’s meetings

We have built upon Haddon et al. (2020) and STC’s (2021) claims of a ‘core group’ of experts by quantifying the number of experts that attended meetings in each timeframe and throughout our study period. It is important to note that the roles of some attendees switched from experts to observers and vice versa during the process—therefore, in our final count, we only accounted for each attendee’s most frequent role. Our analysis found that only 32 of the 142 people we classified as experts attended more than 50% of the meetings (see Table 2). Of these 32, only nine were constantly in the core group, meaning they attended over 50% of meetings in each timeframe. Sixteen experts attended over 50% of meetings in 4 timeframes. Additionally, 63 experts only attended one meeting. We make similar observations for the observers; 39 of the 85 listed attended only one meeting. The number of individuals named and attending meetings increased over our study period, with an average of 15.1 experts attending in TF1, increasing to 38.3 experts in TF5. Moreover, the minutes redacted fewer experts’ names than those we classified as observers or secretariat.

**Table 2:** Meeting attendance and the average number of redacted names over the study period.

Timeframe (TF)	No. of meetings	No. of experts^a^ listed in minutes^b^	No. of experts named and attending 50% or more of meetings^c^	Number of experts named in meetings			Average number of redacted names^d^ in meetings		
				Average	Min.	Max.	Experts	Observers	Secretariat
TF1	16	37	15	15.1	8	21	1.25	1.00	4.63
TF2	23	56	32	28.3	16	37	0.30	2.13	7.26
TF3	19	78	35	29.0	7	47	0.63	6.47	13.21
TF4	16	74	36	34.9	29	42	0.56	7.00	15.94
TF5	15	79	39	38.3	30	44	0.67	8.27	15.20
Whole study period	89	142	32	29.0	7	47	0.65	10.78	4.76

^a^Some attendees switched from experts to observers and vice versa during the process—however, in our final count, we only accounted for each attendee’s most frequent role.

^b^This is our count using our expert database, rather than the official count provided in the minutes. On some occasions, we found that the number of named attendees plus the listed redactions did not add up to the officially provided number of total attendees e.g., meeting 62.

^c^Value taken is rounded up, e.g., if 15 meetings, those listed attended eight or more meetings.

^d^These are the officially provided counts for redactions in the meeting minutes.

**Fig. 3:**
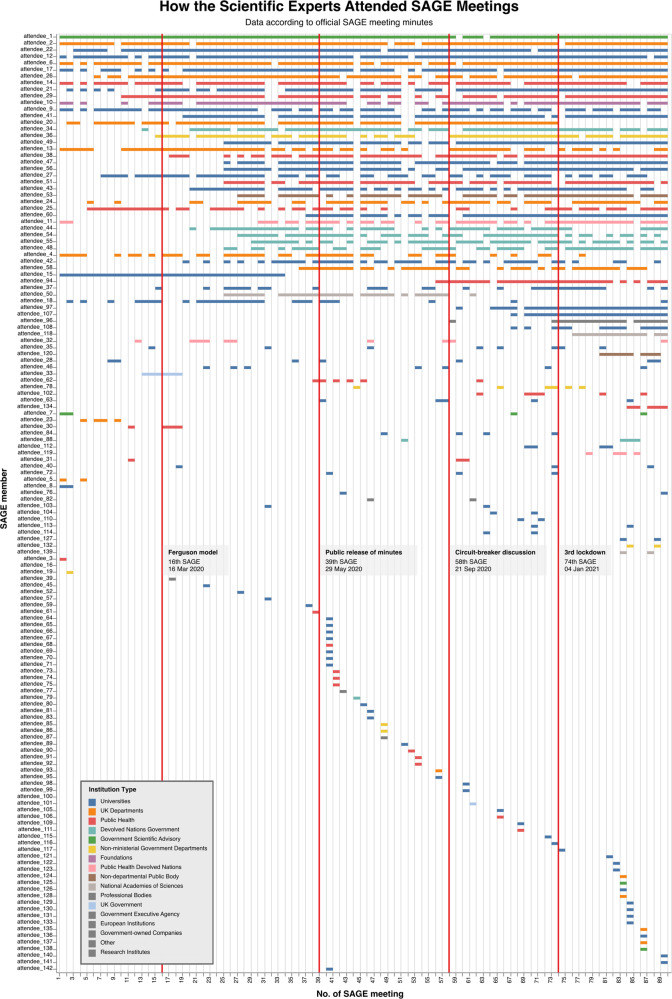
Variation in SAGE experts’ meeting attendance and institution type. Each row represents a different person we have classified as an expert, according to their most frequent role, and the meetings they attended. Institution types are indicated by colour and categorised by us based on information provided on SAGE’s list of participants. Original data source: SAGE Meeting Minutes (1–89) from UK Government (2021b).

The increase in meeting attendance indicates new expertise feeding into SAGE as the pandemic developed. Figure 3 shows this increased attendance, alongside the core group and variation in each participant’s attendance. We also show the institution type of the attendees (our categorisation is available in Supplementary Information 6). Universities, and therefore academics, clearly have a prominent role, alongside representatives from a range of Government bodies and institutions.

Although we classified 142 people listed as experts in the minutes, the number of meetings they attended varies significantly. One concern with the existence of a ‘core group’ is the danger of too few dissenting voices in the science advisory committee and its susceptibility to groupthink (Haddon et al., 2020; STC, 2021).

Now that we have established that during our study period, transparency increased and although there was a core group of experts, there was an increase in the number of people attending SAGE meetings, the following sections move on to our linguistic analysis to answer the second and third research questions.

### The role and attitude of SAGE as constructed on the ‘front stage’

We found that instances where the minutes explicitly reference SAGE provide insights into the portrayal of SAGE’s role. The use of verbs such as “believe”, “endorse”, “view”, “recognise”, “agree”, “think” and “recommend” in the minutes serve to make the stance or position of SAGE explicit within the data. For example, “recognises” is often used to acknowledge constraints with statements, while “recommend” suggests actions to take.

We identified examples explicitly outlining SAGE’s role, including^9^:

“SAGE is responsible for coordinating science advice across [Her Majesty’s Government] HMG, including from [New and Emerging Respiratory Virus Threats Advisory Group] NERVTAG.” (Meeting 2—28 January 2020)

“SAGE reemphasised that its own focus should always be on providing clear scientific advice to government and the principles behind that advice” (Meeting 34—7 May 2020).

In both examples, SAGE’s self-representation as scientific advisors is clear. Furthermore, the minutes also outline what SAGE’s role is not:

“SAGE did not endorse the paper in its current form as SAGE does not give specific operational advice. This is a matter for [Health and Safety Executive] HSE and the safer working place group” (Meeting 34—7 May 2020).

However, these specific minutes also state “SAGE agreed with underpinning scientific principles in the paper”. The presented paper focused on risk assessments for different work activities and settings and highlighted the need to develop operational guidance.

The detailed outline of others’ responsibilities and tasks, e.g., government departments, demarcates responsibility and therefore accountability:

“SAGE also noted the importance of consistent guidance across various settings and sectors: this is the responsibility of [Public Health England] PHE, [Deputy Chief Medical Officers] dCMOs and lead government departments” (Meeting 47—16 July 2020).

Despite these clear delimitations of roles and responsibilities, the scientific findings are sometimes described with the implications of the scientific advice in mind, as discussed by Pamuk (2021). For instance, the minutes may discuss policy choices:

“SAGE would be able to provide an estimate of the number of tests required as part of a contract tracing programme based on the policy mix chosen. SAGE has provided a framework for some of the policy choices” (Meeting 29—28 April 2020).

Building on the tension of usefulness and neutrality (Pamuk, 2021), we investigated the use of attitude markers. The most used attitude markers in the data include: “should”, “important”, “will”, the self-reference construction “SAGE agreed”, and “*would*”. Attitude markers often highlight the shift away from neutrality, conveying attitude and subjective judgement (e.g., “Where the UK does not adopt measures seen in other countries, government should clearly explain its reasoning”, Meeting 15—13 March 2020).

We found that self-reference and attitude markers used in combination highlighted salient judgements contained within the minutes:

“**SAGE advises** that the UK geographical case definition **should** be widened, taking into account available information on air travel volumes from Hubei to other countries, numbers of reported cases in other countries, and understanding of other travel routes.” (Meeting 5—6 February 2020)

“**SAGE advised** that decisions to change alert levels **would** be better based on more than a single criterion or measure; judgements **will** be required, and it is important to consider both relevant data sources and what steps will be required when thresholds are met…” (Meeting 39—28 May 2020)

The collocation of emphatic terms e.g., “importance” and “strongly” alongside SAGE’s self-reference also expressed authority:

“SAGE highlighted the critical **importance** of scaling up antibody serology and diagnostic testing to managing the epidemic.” (Meeting 16—16 March 2020)

“SAGE advised **strongly** that identification of high-risk institutional settings is essential (for example homeless shelters and prisons) and that plans to reduce transmission in these setting must be proactive.” (Meeting 39—28 May 2020)

These examples convey strong affective attitudes towards specific issues and topics. They are signalling SAGE’s stance on certain issues, thus, in our view, not taking a neutral stance.

The meeting minutes explicitly portray SAGE’s role as scientific advisors and the responsibility of government departments and ministers as the policymakers. SAGE Guidance (Cabinet Office, 2012) states that scientific and technical advice on policy choices is likely to be sought. However, we agree with Pamuk (2021) that there is a tension between neutrality and the usefulness of scientific advice. Such tension is particularly evident in the use of attitude markers and SAGE taking a stance on issues.

Usefulness may be maximised by presenting a coordinated viewpoint on issues, whilst still acknowledging any disagreements or uncertainty. For example, Whitty and Collet-Fenson (2021, p. 2) state: “[SAGE] exists to ensure government can integrate science from multiple groups, and that a single version of the science, presented with appropriate levels of confidence and outlier opinion if relevant, is presented to policymakers rather than each department working to a different model”. We now move on to discuss the minutes’ portrayal of consensus and the communication of uncertainty.

### SAGE’s portrayal of consensus and communication of uncertainty

Our analysis of meeting attendance shows numerous people attended SAGE meetings with expertise feeding in and out of discussions, alongside the core group of people consistently attending meetings. Dissenting opinions are therefore likely, as evidenced by Vallance and Whitty multiple times (Haddon et al., 2020, STC, 2021, Woodcock and Buchan, 2020). However, a central view of SAGE is provided through using self-references and attitude markers within the minutes. Therefore, in our interpretation, SAGE is presented unambiguously as a body of consensus.

While we did not identify explicit signposting of disagreement in our sample, i.e., no identified mentions of “disagree” or “do/did/does not agree”, unanimous agreement *is* emphasised, possibly indicating lesser degrees of unanimous agreement when it is not explicitly stated. The minutes use words such as “unanimous” and “completely” to persuasively make a case for, or recommend against, a particular course of action:

*“*SAGE was **unanimous** that measures seeking to **completely** suppress spread of COVID-19 will cause a second peak.” (Meeting 15—13 March 2020)

“There is uncertainty on our exact position, but the **consensus view** is that we are 2–4 weeks behind the epidemic curve in Italy.” (Meeting 17—18 March 2020).

Specific outcomes or foreseen events are portrayed by highlighting the certainty attached to prediction in the text. Pragmatically speaking, the function builds a case of support for a particular course of action.

Linguistic markers indexing the degree of certainty attached to various assertions are common in the meeting minutes. For example, often in sentences pointing to evidence or specific data sources, boosters are used to emphasise the evidential basis for assertions:

“…evidence **shows** that the earlier and more rapidly interventions are put in place, and the more stringent they are, the faster the observed reduction in incidence and prevalence.” (Meeting 69—19 November 2020*)*

“There is still **clear** evidence of the negative educational impact of missing school, **particularly** for younger children (high confidence) …” (Meeting 80—11 February 2021)

In the following example, the use of “will” serves to strengthen the conviction attached to the assessment of how early advice will impact people’s behaviours. Notably, this precedes an advisory statement. The boosters highlight the underpinning rationale, and the confidence of the assessments before issuing advice/guidance.

“Providing advice early would allow people to plan and **will** increase their ability to adhere to guidance. Early advice is **particularly important** in informing people’s travel arrangements. SAGE advises preparing communications on this as soon as possible.” (Meeting 64—29 October 2020)

Conversely, the minutes use hedges to signal when assessments and estimates are not or cannot be delivered with extreme precision or certainty. Sometimes, the text directly points out the inability to be more precise, e.g., a lack of data or evidence or the fallibility of modelling.

“There are **probably** more cases in the UK than SAGE previously expected at this point, and we **may** be further ahead on the epidemic curve, but the UK remains on broadly the same epidemic trajectory.” (Meeting 15—13 March 2020)

“Modelling **indicates** that daily testing of contacts of confirmed cases of COVID-19 **may** offer a supplement or alternative to quarantine strategies.” (Meeting 83—11 March 2021)

As much of the content within the minutes relates to emerging and evolving data, there is an acute need to accurately signal the difference between probable and possible outcomes.

The analysed text introduced explicit labelling strategies (e.g., low, medium, high confidence), as well as marking degrees of certainty (e.g., via boosters and hedges). The summary of Meeting 4 (4 February 2020) states: “Participants were asked to put confidence intervals around statements where possible.” This introduction of a formalised method for encoding the certainty-qualified dimensions of the information contained within the documents demonstrates a commitment to accurately communicating the confidence attached to assessments in this context. However, although the minutes from the fourth meeting suggest using confidence intervals, the practice is employed only sporadically (once in meetings 4, 6, 10 and 14), with more regular (but not consistent) usage emerging from Meeting 31 (1 May 2020) onwards.

In the following sections, we further discuss these changes in the communication of uncertainty, both implicitly using hedges and boosters and explicitly using confidence intervals.

### Frequency and variation of linguistic markers

Our quantitative analysis shows that overall, hedges are more frequently observed in the data than boosters, attitude markers and self-references (see Table 3). That is in line with observations of distributions in the domains of news and academic writing (e.g., Hyland, 2005; Shen and Tao, 2021). However, our research focuses on how the frequencies vary over time (also shown in Table 3).

**Table 3:** Frequencies of self-references, attitude markers, boosters, and hedges per 1000 words over the study period.

TFs	Self-references		Attitude markers		Boosters		Hedges	
	Mean	Standard deviation	Mean	Standard deviation	Mean	Standard deviation	Mean	Standard deviation
1	7.09	4.12	12.92	10.12	13.36	6.31	25.46	8.11
2	7.27	3.2	10.5	6.68	10.12	4.46	22.69	7.88
3	7.24	3.02	10.21	5.26	13.69	5.14	25.81	7.38
4	3.83	1.36	6.00	3.19	15.72	4.02	33.64	6.23
5	1.20	0.95	8.18	3.95	16.48	4.79	32.97	5.15
All data	5.52	3.64	9.60	6.42	13.82	5.34	27.85	8.12

Supplementary Information 4 provides a complete list of markers, alongside frequencies.

**Fig. 4:**
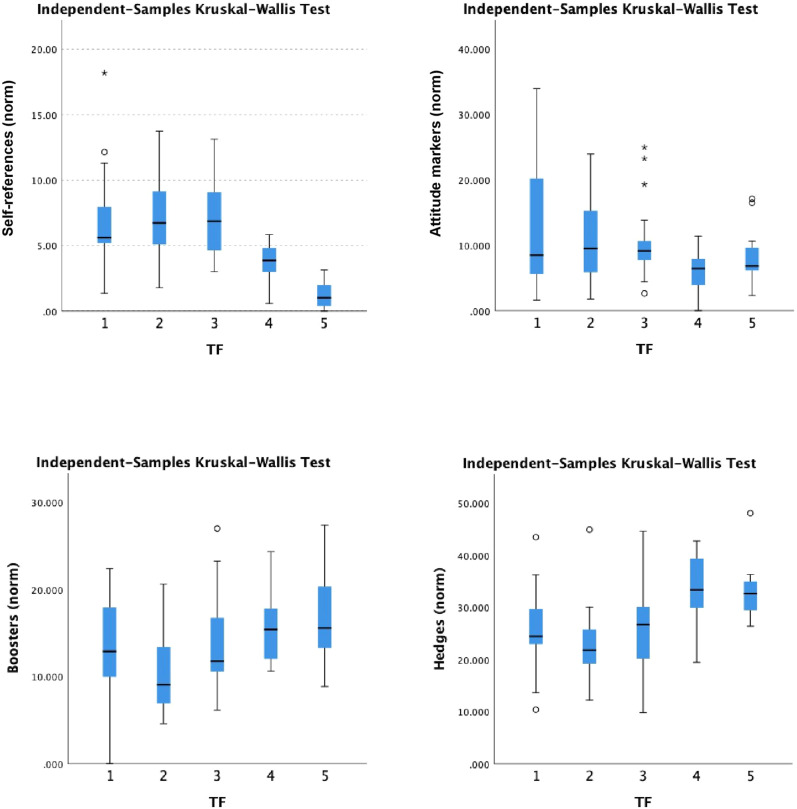
Boxplots of marker frequencies per 1000 words in each timeframe. Outliers are identified using small circles to denote ‘out’ values and stars to denote ‘far out’ values (referred to as ‘extreme values’ in SPSS).

A non-parametric independent samples Kruskal–Wallis test (Fig. 4) confirms a statistically significant (i.e. a *p*-value < 0.05) variation of frequency of self-references across timeframes (*H*(4) = 48.419, *p* < 0.001), with pairwise comparison demonstrating significantly fewer self-references used in TF5 than TF1 (*p* < 0.001), TF2 (*p* < 0.001) and TF3 (*p* < 0.001), as well as fewer in TF4 than TF2 (*p* = 0.025) and TF3 (*p* < 0.011). There was no significant variation between any other pairwise comparisons. In summary, fewer self-references feature in the latter compared to earlier timeframes. Although there is a slight downward trend in the use of attitude markers, there is no statistically significant variation across timeframes (*H*(4) = 9.069, *p* = 0.059).

The frequency of hedges varies significantly across timeframes (*H*(4) = 29.001, *p* < 0.001). Post-hoc pairwise comparisons with Bonferroni correction shows there are significantly more hedges in TF4 compared to TF1 (*p* = 0.029), TF2 (*p* < 0.001) and TF3 (*p* = 0.014), and TF5 compared to TF1 (*p* = 0.036), TF2 (*p* < 0.001), and TF3 (*p* = 0.018). No significant difference was observed between TF4 and TF5 (*p* = 1.00), nor were any other comparisons. In summary, the latter timeframes use more hedges, significantly so from TF4 onwards.

Frequencies of boosters across the five timeframes vary significantly (*H*(4) = 15.845, *p* = 0.003), with pairwise comparison demonstrating significantly fewer boosters were used in TF2 than TF4 (*p* = 0.011) and TF5 (*p* = 0.004). There were no other significant variations across timeframes. In summary, markedly fewer boosters are used in TF2 than in later timeframes.

We also analysed the mentions of confidence intervals in the minutes. As previously noted, the minutes did not express these consistently. There are 405 instances in the data, with a mean value of 4.55 per meeting (min: 0, max: 42, standard deviation: 7.52), but, for example, in meeting 84 held on 25 March 2021, there are 42 instances. High confidence is the most used interval accounting for 52.59% of all confidence intervals, followed by medium or moderate (33.58%), with low and variations such as very low accounting for only 13.83%. Explicit confidence intervals, when used, are more commonly employed to emphasise higher levels of certainty.

We now discuss what these changes imply about the communication of uncertainty and the portrayal of SAGE’s role.

### Changes in the communication of uncertainty and the portrayal of SAGE’s role

Our quantitative analysis shows that the frequency of hedges increased through our study period. Meeting minutes from TF2 have less than half as many boosters as TF4 and TF5. This observation is perhaps less surprising when we consider the events in TF2—the period when the virus first emerged in the UK, with the potential scale of the pandemic becoming more apparent and leading to a national lockdown. At that time, the lack of available scientific evidence was most pronounced, and there were calls for more data within the meeting minutes. It is plausible that making strong claims was a more challenging and precarious endeavour. Conversely, in TF4 and TF5, the need to convey assessments with a conviction may have been increasingly important, hence the increase in boosters.

SAGE advised a ‘circuit-breaker’ lockdown for the first time at the end of TF3 and reiterated this advice in TF4. Within TF4-5, SAGE also expressed concerns about the winter period and Christmas festivities (e.g., meeting 66, 5 November 2020) and discussed the national lockdown in 2021. As the earlier examples highlight, hedges forefront the uncertainty associated with particular claims. They also make apparent what it is not possible to yet know definitively. This suggests that greater caution was employed as the pandemic progressed, with greater use of hedges to denote a lack of certainty or a decrease of authority in particular claims. As the situation developed and the availability of information and relevant data improved, it is understandable that the ability and willingness to assert statements more confidently or with higher degrees of certainty using boosters would increase. However, with increased media coverage, the importance of precisely defining caveats or limitations could explain the simultaneous and statistically significant increase in hedges. This reasoning suggests that SAGE was particularly and increasingly cautious about flagging what was not yet known, subject to change, or reliant on further data.

The following example, taken from the penultimate meeting in TF5, features the booster “highly” jointly used with the hedges “likely” and “uncertain”.

“It remains **highly likely** that there will be a further resurgence in hospitalisations and deaths at some point, however, the scale, shape, and timing remain **highly uncertain***.*” (Meeting 88—5 May 2021)

In combination, hedges and boosters are employed to demonstrate attentiveness to the importance of marking confidence in assessments. Therefore, the increase in these features points to a rising effort to be precise and explicit about the probabilities and confidence attached to assertions as the pandemic progresses. The increasing use of explicit confidence intervals further supports this interpretation.

In contrast, self-references and attitude markers decrease. We compared the keywords characterising the sentences of SAGE self-representation to investigate this change further (see Supplementary Information 3). In TF1-3, only two words are significantly more common compared to TF4-5—“agreed” and “but”. “Agreed” justifies a position, pointing to a ‘backstage’ rationalisation and reasoning process. The other keyword, “but”, is a contrastive maker, and it again is used when other possibilities, alternatives and considerations are factored into the statement. Both keywords demonstrate the consensus-building process of deliberation underpinning the conclusions put forward and potentially for dissenting views.

In the latter stages, when more knowledge about the virus had been established, SAGE’s position on specific items had already been provided, so re-emphasising and referring to the prior judgements dominates. Consequently, in TF4-5, the keywords relate to signposting previously stated positions (e.g., “previously” and “has”, as in “has previously advised”, and “see”, which is used to reference specific past meetings, for example, “see SAGE 46”). As the self-references serve as a commitment to knowledge claims and authorial responsibility, one interpretation for the decrease over time is that SAGE had already established a stance on key topics. An alternative explanation is that SAGE was taking a more cautious approach, i.e., creating distance between themselves and the policy decisions, and more fully accounting for degrees of certainty, as shown through the increase in boosters and hedges.

The analysis demonstrates how the meeting minutes depict changes in SAGE’s approach to transparency, stance, and uncertainty. The use of certainty and stance markers over the period studied reveals shifts in how scientific ambiguity was reported and how SAGE’s advisory position was communicated. We also confirmed that there was a ‘core group’ of experts. Going back to Hilgartner’s (2000) ‘Science on Stage’ metaphor, the next section of this paper considers what these changes may mean for SAGE’s future ‘front stage’ performance.

### SAGE’s future ‘front stage’ performance

In this paper, we have drawn together information from secondary sources (including Haddon et al., 2020; STC, 2021) and analysed the meeting minutes to explore: (1) Whether SAGE’s approach towards transparency and a plurality of expertise changed throughout the study period; (2) How SAGE’s role was constructed through the meeting minutes; (3) How consensus and uncertainty were communicated within SAGE’s meeting minutes.

The answer to our first research question provides a ‘backdrop’ for the second and third questions. We have substantiated the claims that SAGE’s level of transparency increased by releasing the minutes and the experts’ names (UK Government, 2020a). As stated by both Haddon et al. (2020) and STC (2021), there was a core group of people consistently attending SAGE meetings (Table 2 and Fig. 3), but we found that the average number of people attending did increase across our timeframes. We contend that although the process has become more transparent, a clearer picture of the specific expertise feeding into discussions would further improve transparency. On SAGE’s website, for example, the single list of participants currently conflates those in the core group by listing them alongside those who have only attended a few meetings (Clark, 2020).

Turning to the second research question, the phrase (and variations of) “following the science” drew attention to the contested boundary between science and politics (Gieryn, 1983; Jasanoff, 1990, 1987) through the staging of scientific experts to legitimise claims (Kettell and Kerr, 2022; STC, 2021). However, the science and policy boundary is not always clear in decision-making. We found that the minutes foregrounded SAGE’s role as science advisors, and there are examples of the responsibilities and, therefore, accountability of others outlined, e.g., Government departments referred to as policymakers. However, although scientists advise and ministers decide (Atkinson et al., 2020; Clark, 2020), we argue that attitude markers demonstrate that SAGE takes a stance on issues, while also discussing policy choices (which is part of their remit according to their Guidance—see Cabinet Office, 2012). Against the background of Pamuk’s (2021) work on neutrality versus usefulness, SAGE seemingly takes a more ‘useful approach’. Furthermore, over our study period, we identified a decrease in attitude markers and self-references to SAGE. One possible explanation for this reduction is fewer rapid scientific developments in the latter stage of our study period. Keywords such as “*previously*” also indicate more signposting to former statements made or reiterating SAGE’s stance on particular topics. Alternatively, SAGE may have been taking a more cautious approach (also shown through the increase in uncertainty markers) and safeguarding their credibility and reputation by detaching themselves from policy decisions (Metcalfe et al., 2020).

Our third research question addresses the themes of communicating uncertainty and consensus. Although (on average) the number of voices feeding into discussions increased (as established in the first research question) and thus it is likely there were more perspectives feeding into discussions, in our sample of minutes SAGE presented a consensus view—therefore aggregating these experts’ views as a unified voice. This portrayal of consensus has been criticised during the COVID-19 crisis and in previous SAGE events (Haddon et al., 2020; STC, 2021). The criticisms frequently called for greater openness about disagreement (Moore and MacKenzie, 2020). This type of openness is also a recommendation in SAGE’s own Terms of Reference (Cabinet Office, 2012). Providing universal authoritative advice is also at tension with an expectation of transparency on how such a position is formed. Wording such as “agreed” and “discussed”, and contrastive markers such as “but” indicate SAGE’s discursive practice within meetings and potentially dissenting views. However, in our sample, we found little evidence of explicit signposting of disagreement. Pamuk (2021) has suggested following the best practice of the US Supreme Court and publishing dissenting opinions to avoid backstaging the inevitable deliberation process of any committee. This suggestion aligns with CoPSAC 2021 (Government Office for Science, 2021, p. 21), which states “Whilst achieving consensus should be the objective, where this is not possible the record should include the majority and minority positions, explaining the differences and reasons for them”.

Lastly, although the marking of uncertainty is also encouraged in the Guidance (Cabinet Office, 2012), in the pandemic’s early stages, explicit markers (and implicit) are limited compared to the latter stages. The increased use of linguistic markers for certainty and uncertainty, i.e., hedges and boosters, indicates a growing commitment to the importance of being precise about the known and unknown ambiguities in the underlying science. This change in approach potentially reflects a learning curve of SAGE. In the early stages of the pandemic, COVID-19 was a new and unfamiliar threat with several unknowns (Balog-Way and McComas, 2020). Despite the use of markers increasing, we argue this does not necessarily imply a rise in uncertainty. Instead, it reflects greater confidence about the dimension of uncertainty. The introduction of explicit uncertainty through confidence intervals, which the minutes encouraged early on (SAGE meeting 4, 4 February 2020), further supports this argument. As with the notion of consensus, this increased awareness of communicating or even implicitly expressing uncertainty informs the public’s understanding of scientific ambiguity, and we think it should form part of future guidance on constructing meeting minutes.

## Conclusions and limitations

Our study focused on the meeting minutes over a fixed study period. The pandemic continued beyond this, and the emergence of the Omicron variant in November 2021 highlighted scientific uncertainties and developments in scientific understanding remained. The reported leaking of SAGE’s minutes by traditional media outlets (Forrest, 2021; Reed, 2021) foregrounded the continued public interest in SAGE. This leaking of the minutes raises further questions about who the final decision-makers and influencers on transparency decisions are, including the release date— as well as demonstrating the minutes are a key first-hand account of SAGE’s meetings and the current scientific advice. The Government Office for Science should therefore discuss, and potentially refine, the protocols for their minutes’ construction, as they could again become a pacemaker for the public discussion of emergency policies in future SAGE activation.

This paper has not addressed other aspects of SAGE’s ‘front stage performance’ beyond the meeting minutes—particularly the representation of SAGE during the COVID-19 public press briefings. Additionally, we have analysed a sample of the minutes and have not analysed other documents linked to or referred to in the minutes. For example, there are ‘consensus statements’ for various sub-committees (which refer to language frameworks for discussing probabilities—see SPO-M-O, 2021) and academic papers that have fed into discussions. Furthermore, our exploration of the ‘core group’ concept could be extended by analysing the experts’ disciplines, and if the topics discussed fell within the remit of SAGE and these experts’ judgement. SAGE’s website, nor the minutes, did not provide the expert’s disciplines, therefore, we did not include them in our analysis. Analysing the topics was outside the scope of this investigation.

This research is not a public enquiry, nor does it intend to apportion blame or credit (also see Birch, 2021). We had to assume that the SAGE minutes accurately reflect the scientific advice provided to COBR and other Government Departments. Accordingly, the minutes are not likely to capture informal conversations potentially feeding into policy decisions (Birch, 2021). The minutes are crafted documents. Those writing the minutes make value judgements about what to include (or not) (Elliott, 2020); these judgements are likely to vary depending on the author. In our sample, there was no indication of whether the minute taker(s) changed. If they did, this might explain discrepancies from meeting to meeting in the linguistic choices, e.g., some meetings with higher levels of confidence intervals expressed than others. We have addressed this limitation by using timeframes and looking at overall trends in the data. Furthermore, the process of writing the minutes is also backstage—e.g., to what extent the initial notes taken at the meeting were refined until the release into the public domain. The SAGE secretariat is responsible for writing the meeting minutes (Cabinet Office, 2012), and from the publicly available documents, it is impossible to know if all the individual experts’ views are reflected and how these were aggregated. Like Hilgartner’s (2000, 2004) and Takahashi (2019) research, the analysis is restricted to published materials and archival documents. Ethnographic observation would be needed to understand the whole process accurately. Without talking to SAGE experts or observing the meetings, we also do not know the impact of external influences on SAGE’s evolvement, such as the establishment of ‘Independent SAGE’^10^, who were set up with transparency of scientific advice as one of their overarching principles (see Atkinson et al., 2020; Jasanoff et al., 2021; Clarke, 2021; Horton, 2020; Inge, 2020). By highlighting these limitations, our research emphasises how much the public did not have access. Within our study period, there continued to be an opaqueness, including the deliberations that took place during the meetings and how the scientific advice was translated into policy decisions (Haddon et al., 2020; STC, 2021). As highlighted by Elliott (2021) and Nguyen (2021) there are potential ‘dangers’ with the level of transparency which results in a practical dilemma with no neat resolution. However, even if aspects remain ‘backstage’, those making these transparency decisions should clarify their justifications.

In conclusion, throughout our study period, SAGE diverged from its usually strictly temporary setup to become a quasi-permanent institution at the heart of the UK’s response to the COVID-19 pandemic (Haddon et al., 2020). By design, SAGE is an ad hoc group but had to adapt to both acute and long-term demands. In crises, such as COVID-19, decisions on transparency, a plurality of expertise, and the articulation of roles, responsibilities, consensus, and uncertainties become more pressing. We have demonstrated how the meeting minutes depict changes in SAGE’s approach to transparency, stance and uncertainty and we highlighted how linguistic analysis can be a useful approach for developing our understanding of science communication practices and scientific ambiguity.

## Supplementary information

Supplementary Information

## Data Availability

The data generated or analysed during this study are included in this published article and its supplementary information file under the following headings: Supplementary Information 1: Google Trends Data for SAGE in the UK. Supplementary Information 2: SAGE Timeline and Corpus Review of Media. Supplementary Information 3: SAGE Self-References. Supplementary Information 4: Marker Frequency Data. Supplementary Information 5: SAGE meeting minutes—Time of Release. Supplementary Information 6: People attending SAGE meetings.

## Footnotes

1 We queried https://trends.google.co.uk/ for the search terms ‘sage government’, ‘sage covid’ and ‘sage minutes’ for the time range 1 January 2019 to 31 May 2021. We compared this with Google’s own created search topic ‘Scientific Advisory Group for Emergencies’, defined as “group of terms that share the same concept in any language” (Google, 2022). Both share the same baseline trend, however, exploring the interest over time in the topic yields a greater relative interest from mid-April to late May 2020 and less pronounced peaks thereafter (see Supplementary Information 1).

2 Within CoPSAC 2021 it states: “The Scientific Advisory Group for Emergencies (SAGE) provides advice to support government decision makers during emergencies when activated. It is not a scientific advisory committee or council in a formal sense. SAGE has no membership. Its participants are called upon depending on the nature of the emergency and the issues under consideration change from meeting to meeting. While alignment with the CoPSAC is sought as far as practical, several sections of the CoPSAC do not directly apply (although for some emergencies, SAGE may work with existing SACs which are covered by CoPSAC). SAGE’s guiding documents are published here.” (Government Office for Science, 2021). ‘Here’ directs the reader to SAGE’s website, specifically the ‘About Us’ section (UK Government, 2021a) which contains SAGE’s ‘Terms of Reference’ (see Cabinet Office, 2012).

3 All meeting minutes are available from UK Government (2021b). We have not provided URL references for each individual meeting. Instead, the meeting minutes are referenced using the meeting’s number and date.

4 Additional people have been added to SAGE’s website list after our study period.

5 The filtering of the self-reference data was completed in the corpus analysis tool Sketch Engine (Kilgarriff et al., 2014).

6 Keywords were identified using the corpus analysis software Antconc (Anthony, 2021).

7 The concordance view in Sketch Engine (Kilgarriff et al., 2014) was used to generate the initial list of markers (building on Shen and Tao, 2021) and to manually inspect the use of each marker.

8 Final counts for agreement between researchers: “will”, 99.3% (*n* = 752); “would”, 96.92% (*n* = 389); “must”, 90% (*n* = 20).

9 Within the analysis, we provide evidence, i.e., excerpts from the minutes exemplary for our different aspects of analysis where all emphasis (inc. bold text and acronyms expanded as square brackets) in the quotations is ours.

10 Independent SAGE (2021) is a group of scientists publishing scientific advice relevant to COVID-19. They are unaffiliated with the Government, although some members are also on the official SAGE. The group was founded by former Government CSA, Sir David King. King warned that the perceived lack of transparency and Ministers’ claims they were ‘following the science’ could cause a loss of public trust (Devlin, 2020).
