# Supplementary material for: COVID-19 and science advice on the ‘Grand Stage’: the metadata and linguistic choices in a scientific advisory groups’ meeting minutes
Source: Humanit Soc Sci Commun. 2022 Dec 24;9(1):465. doi: 10.1057/s41599-022-01403-1 (PMC9789727; doi:10.1057/s41599-022-01403-1)
Supplement: Supplementary file 1 — Supplementary Information [file 41599_2022_1403_MOESM1_ESM.pdf]

Supplementary Information:

COVID-19 and Science Advice on the 'Grand Stage': Metadata and Linguistic Choices in a Scientific Advisory Groups' Meeting Minutes

Hannah Baker[1*], Shauna Concannon[1], Matthias Meller[2], Katie Cohen[1], Alice Millington[1], Samuel Ward[1] & Emily So[1].

[1] University of Cambridge, UK.

[2] The Technical University of Munich, Germany.

*corresponding author. Email: heb51@cam.ac.uk

	Page Number
**Supplementary Information 1: Google Trends Data for SAGE in the UK**	**3**
**Supplementary Information 2: SAGE Timeline and Corpus Review of Media**	**7**
SAGE Timeline and Key Events	8
Reference data for corpus review of media	10
**Supplementary Information 3: SAGE Self-References**	**12**
SAGE self-reference frequencies	13
SAGE self-references keyword comparison	15
**Supplementary Information 4: Marker Frequency Data**	**16**
Total frequencies of markers per meeting	17
Boosters’ frequency overview	19
Hedges’ frequency overview	21
Attitude markers’ frequency overview	23
**Supplementary Information 5: SAGE meeting minutes - Time of release**	**24**
**Supplementary Information 6: People attending SAGE meetings**	**30**
Institution Categories	31
Redacted name count	34

Supplementary Information 1: Google Trends Data for SAGE in the UK

The following pages record the reference data from Google Trends (https://trends.google.co.uk) on searches for SAGE in the UK.

Trends	Description
Search Term Comparison: 'sage' + '...'	Three-way comparison of the relative interest in search terms 'sage government', 'sage covid' and 'sage minutes'
Search Topic: 'Scientific Advisory ...'	Relative interest in the search topic 'Scientific Advisory Group for Emergencies'

Methodology

We queried https://trends.google.co.uk/ for the search terms ‘sage government’, ‘sage covid’ and ‘sage minutes’ for the time range 01 January 2019 to 31 May 2021 in the United Kingdom. We compared this with Google’s own created search topic ‘Scientific Advisory Group for Emergencies’, defined as a “group of terms that share the same concept in any language” (Google, 2022; https://support.google.com/trends/answer/4359550?hl=en, accessed 17 June 2022). Google Search had a market share of 86.31 per cent in the UK as of 2021 (see http://www.statista.com/statistics/280269/market-share-held-by-search-engines-in-the-united-kingdom/), so Google Trends arguably provides an indicator of the public interest of a search term.

Accessed: 17 June 2022

Search Term Comparison: 'sage' + 'government/covid/minutes'

Week	sage government: (United Kingdom)	sage covid: (United Kingdom)	sage minutes: (United Kingdom)
**2019-01-06**	1	0	0
**2019-01-13**	1	0	0
**2019-01-20**	1	0	0
**2019-01-27**	0	0	0
**2019-02-03**	0	0	0
**2019-02-10**	1	0	0
**2019-02-17**	3	0	1
**2019-02-24**	0	0	2
**2019-03-03**	0	0	0
**2019-03-10**	3	0	0
**2019-03-17**	0	0	0
**2019-03-24**	1	0	0
**2019-03-31**	1	0	0
**2019-04-07**	1	0	1
**2019-04-14**	0	0	0
**2019-04-21**	1	0	0
**2019-04-28**	1	0	0
**2019-05-05**	1	0	1
**2019-05-12**	1	0	0
**2019-05-19**	1	0	0
**2019-05-26**	0	0	0
**2019-06-02**	0	0	0
**2019-06-09**	2	0	1
**2019-06-16**	1	0	0
**2019-06-23**	1	0	1
**2019-06-30**	1	0	0
**2019-07-07**	2	0	1
**2019-07-14**	1	0	0
**2019-07-21**	5	0	0
**2019-07-28**	1	0	0
**2019-08-04**	3	0	0
**2019-08-11**	1	0	0
**2019-08-18**	1	0	0
**2019-08-25**	1	0	0
**2019-09-01**	1	0	0
**2019-09-08**	4	0	0
**2019-09-15**	1	0	0
**2019-09-22**	1	0	1
**2019-09-29**	2	0	1

Cont. this page

Week	sage government: (United Kingdom)	sage covid: (United Kingdom)	sage minutes: (United Kingdom)
**2019-10-06**	0	0	0
**2019-10-13**	2	0	0
**2019-10-20**	1	0	1
**2019-10-27**	2	0	0
**2019-11-03**	4	0	0
**2019-11-10**	1	0	0
**2019-11-17**	1	0	0
**2019-11-24**	0	0	1
**2019-12-01**	0	0	0
**2019-12-08**	1	0	0
**2019-12-15**	3	0	0
**2019-12-22**	0	0	0
**2019-12-29**	1	0	0
**2020-01-05**	1	0	0
**2020-01-12**	1	0	0
**2020-01-19**	5	0	0
**2020-01-26**	1	0	0
**2020-02-02**	1	0	0
**2020-02-09**	3	0	0
**2020-02-16**	4	0	1
**2020-02-23**	3	0	0
**2020-03-01**	2	0	0
**2020-03-08**	3	4	0
**2020-03-15**	11	16	0
**2020-03-22**	9	20	0
**2020-03-29**	7	9	0
**2020-04-05**	31	25	0
**2020-04-12**	100	38	1
**2020-04-19**	78	28	7
**2020-04-26**	55	23	8
**2020-05-03**	37	25	1
**2020-05-10**	26	28	4
**2020-05-17**	33	26	2
**2020-05-24**	35	27	26
**2020-05-31**	18	17	12
**2020-06-07**	19	26	8
**2020-06-14**	12	18	8
**2020-06-21**	18	15	6
**2020-06-28**	9	15	4

Cont. next page

**2020-07-05**	11	17	1
**2020-07-12**	8	15	1
**2020-07-19**	5	7	1
**2020-07-26**	6	13	3
**2020-08-02**	4	13	6
**2020-08-09**	3	13	4
**2020-08-16**	7	12	2
**2020-08-23**	3	10	1
**2020-08-30**	3	5	2
**2020-09-06**	6	19	2
**2020-09-13**	5	18	1
**2020-09-20**	3	19	3
**2020-09-27**	4	13	2
**2020-10-04**	4	17	2
**2020-10-11**	18	40	14
**2020-10-18**	11	27	3
**2020-10-25**	20	42	6
**2020-11-01**	14	20	1
**2020-11-08**	7	13	3
**2020-11-15**	6	14	2
**2020-11-22**	9	27	2
**2020-11-29**	5	18	2
**2020-12-06**	7	17	3
**2020-12-13**	5	24	1
**2020-12-20**	7	25	4
**2020-12-27**	15	43	5
**2021-01-03**	9	54	6
**2021-01-10**	4	27	2
**2021-01-17**	5	21	8
**2021-01-24**	6	22	3
**2021-01-31**	9	16	2
**2021-02-07**	7	22	2
**2021-02-14**	5	19	3
**2021-02-21**	8	33	4
**2021-02-28**	2	13	1
**2021-03-07**	5	8	3
**2021-03-14**	4	12	1
**2021-03-21**	6	14	1
**2021-03-28**	5	12	2
**2021-04-04**	6	24	5
**2021-04-11**	3	11	2
**2021-04-18**	6	18	3
**2021-04-25**	1	20	3
**2021-05-02**	3	18	1

Cont. this page

**2021-05-09**	4	25	3
**2021-05-16**	8	15	4
**2021-05-23**	6	13	3
**2021-05-30**	4	26	4
**2021-05-09**	4	25	3

Search Topic: 'Scientific Advisory Group for Emergencies'

Week	Scientific Advisory Group for Emergencies: (United Kingdom)
**2019-01-06**	0
**2019-01-13**	0
**2019-01-20**	0
**2019-01-27**	0
**2019-02-03**	0
**2019-02-10**	0
**2019-02-17**	0
**2019-02-24**	1
**2019-03-03**	1
**2019-03-10**	0
**2019-03-17**	0
**2019-03-24**	0
**2019-03-31**	0
**2019-04-07**	0
**2019-04-14**	0
**2019-04-21**	0
**2019-04-28**	0
**2019-05-05**	0
**2019-05-12**	0
**2019-05-19**	0
**2019-05-26**	1
**2019-06-02**	0
**2019-06-09**	1
**2019-06-16**	1
**2019-06-23**	0
**2019-06-30**	0
**2019-07-07**	0
**2019-07-14**	0
**2019-07-21**	0
**2019-07-28**	0
**2019-08-04**	0
**2019-08-11**	0
**2019-08-18**	0
**2019-08-25**	0
**2019-09-01**	0
**2019-09-08**	0
**2019-09-15**	0
Cont. this page	

Week	Scientific Advisory Group for Emergencies: (United Kingdom)
**2019-09-22**	1
**2019-09-29**	1
**2019-10-06**	0
**2019-10-13**	0
**2019-10-20**	0
**2019-10-27**	0
**2019-11-03**	1
**2019-11-10**	0
**2019-11-17**	1
**2019-11-24**	0
**2019-12-01**	0
**2019-12-08**	0
**2019-12-15**	0
**2019-12-22**	0
**2019-12-29**	0
**2020-01-05**	0
**2020-01-12**	0
**2020-01-19**	1
**2020-01-26**	0
**2020-02-02**	1
**2020-02-09**	<1
**2020-02-16**	0
**2020-02-23**	1
**2020-03-01**	2
**2020-03-08**	4
**2020-03-15**	38
**2020-03-22**	11
**2020-03-29**	5
**2020-04-05**	40
**2020-04-12**	85
**2020-04-19**	70
**2020-04-26**	64
**2020-05-03**	100
**2020-05-10**	44
**2020-05-17**	59
**2020-05-24**	86
**2020-05-31**	44
Cont. this page	

Week	Scientific Advisory Group for Emergencies: (United Kingdom)
**2020-06-07**	34
**2020-06-14**	19
**2020-06-21**	27
**2020-06-28**	12
**2020-07-05**	11
**2020-07-12**	15
**2020-07-19**	10
**2020-07-26**	13
**2020-08-02**	10
**2020-08-09**	9
**2020-08-16**	8
**2020-08-23**	6
**2020-08-30**	6
**2020-09-06**	11
**2020-09-13**	11
**2020-09-20**	14
**2020-09-27**	6
**2020-10-04**	13
**2020-10-11**	40
**2020-10-18**	18
**2020-10-25**	43
**2020-11-01**	18
**2020-11-08**	7
**2020-11-15**	11
**2020-11-22**	18
**2020-11-29**	8
**2020-12-06**	10
**2020-12-13**	14
**2020-12-20**	14
**2020-12-27**	33
**2021-01-03**	34
**2021-01-10**	10
**2021-01-17**	14
**2021-01-24**	10
**2021-01-31**	9
**2021-02-07**	14
**2021-02-14**	12
Cont. this page	

Week	Scientific Advisory Group for Emergencies: (United Kingdom)
**2021-02-21**	14
**2021-02-28**	8
**2021-03-07**	5
**2021-03-14**	3
**2021-03-21**	7
**2021-03-28**	6
**2021-04-04**	16
**2021-04-11**	3
**2021-04-18**	6
**2021-04-25**	5
**2021-05-02**	4
**2021-05-09**	33
**2021-05-16**	14
**2021-05-23**	9
**2021-05-30**	9

Supplementary Information 2: SAGE Timeline and Corpus Review of Media

The following pages record the reference data and the corpus of reviewed media articles for the timeline of key events and headlines identified in Figure 1.

SAGE Timeline and Key Events

Timeline of key events and headlines identified, based on a media review of The Guardian, The New York Times and the Financial Times between January 2020 and May 2021.

Event type	'Event title' displayed on timeline (Fig.1)	Date	Source	Source title	Corresponding SAGE meeting no.	Corresponding SAGE meeting date
**SAGE meeting**	**SAGE 1st 'meeting**	2020-01-22	UK Government	Precautionary SAGE 1 minutes: Coronavirus (COVID-19) response, 22 January 2020	1	2020-01-22
**Publication**	**New epidemic modelling by Prof Ferguson**	2020-03-17	The New York Times	Behind the Virus Report That Jarred the U.S. and the U.K. to Action	16	2020-03-16
**Publication**	**First lockdown in UK**	2020-03-23	The New York Times	Britain Placed Under a Virtual Lockdown by Boris Johnson	18	2020-03-22
**Publication**	**The "Dominic Cummings on SAGE" affair**	2020-04-24	The Guardian	Revealed: Cummings is on secret scientific advisory group for Covid-19	29	2020-04-28
**Publication**	**Seeking wider expertise for SAGE**	2020-04-29	The Guardian	Government rushes out request for experts to work with Sage panel	30	2020-04-30
**Publication**	**UK Government reveals SAGE members**	2020-05-04	The Guardian	Government names dozens of scientists who sit on Sage group	32	2020-05-01
**Publication**	**Independent SAGE emerges**	2020-05-04	The Guardian	Rival Sage group says Covid-19 policy must be clarified	33	2020-05-05
**Announcement**	**First publication of SAGE minutes**	2020-05-29	UK Government	SAGE: Coronavirus response - publication scheme	39	2020-05-28
**Publication**	**The "Back to Work" affair**	2020-07-16	Financial Times	Johnson suffers 'back to work' blow from chief scientist	46	2020-07-16

Event type	'Event title' displayed on timeline (Fig.1)	Date	Source	Source title	Corresponding SAGE meeting no.	Corresponding SAGE meeting date
**Publication**	**SAGE members urgently call for restrictions**	2020-09-23	Financial Times	Further restrictions needed to halt Covid spreading in UK, scientists warn	58	2020-09-21
**Publication**	**"PM Johnson splits from SAGE" affair**	2020-10-13	Financial Times	Johnson gambles by splitting from his scientists	58	2020-09-21
**Publication**	**"PM Johnson splits from SAGE" affair (cont'd)**	2020-10-13	The Guardian	Sage documents show how scientists felt sidelined by economic considerations	58	2020-09-21
**Publication**	**SAGE and the Christmas controversy**	2020-11-25	The Guardian	Covid-19 infections 'could easily double' over Christmas, Sage experts say	69	2020-11-19
**Publication**	**Lockdown No. 3 announced**	2021-01-04	Financial Times	Johnson bows to the inevitable after surge in Covid cases	75	2021-01-07

Reference data for corpus review of media

The following list is the reference data and corpus of reviewed media articles for Figure 1.

The editorial board, 2020. Even in a pandemic, politicians must decide. Financial Times.

Bosely, S., 2021. Scientists reject Cummings' claim that their advice delayed first UK lockdown. The Guardian.

Bosely, S., 2020. Sage documents show how scientists felt sidelined by economic considerations. The Guardian.

Boyd, I., 2020. I'm proud to be a scientist in Sage – to call us "secretive" is unjustified | Ian Boyd. The Guardian.

Cameron-Chileshe, J., 2020. Boris Johnson to outline Christmas Covid rules. Financial Times.

Cameron-Chileshe, J., Parker, G., 2020. Scientists highly critical of UK test-and-trace, Sage documents show. Financial Times.

Carrell, S., Pegg, D., Lawrence, F., Lewis, P., Evans, R., Conn, D., Davies, H., Proctor, K., 2020. Revealed: Dominic Cummings on secret scientific advisory group for Covid-19. The Guardian.

Clark, P., 2020. Neil Ferguson, a virus modeller sounds the alarm. Financial Times.

Cookson, C., Burn-Murdoch, J., 2021. UK Covid lockdown starting to work, say scientists. Financial Times.

Costello, A., 2020. The government's secret science group has a shocking lack of expertise | Anthony Costello. The Guardian.

Davis, N., 2020a. Rival Sage group says Covid-19 policy must be clarified. The Guardian.

Davis, N., 2020b. Covid-19 infections "could easily double" over Christmas, Sage experts say. The Guardian.

Davis, N., 2020c. 'Kamikaze': the experts urging UK to rethink Christmas Covid rules. The Guardian.

Giles, C., Cameron-Chileshe, J., Cookson, C., 2020. Official figures suggest UK Covid spread was stabilising in October. Financial Times.

Gross, A., Cameron-Chileshe, J., Bounds, A., 2020a. Further restrictions needed to halt Covid spreading in UK, scientists warn. Financial Times.

Gross, A., Cameron-Chileshe, J., Raval, A., Neville, S., 2020b. Second national lockdown proposed by UK scientific advisers. Financial Times.

Hodgson, C., 2020. Scientists call on UK to rethink 'dangerous' coronavirus strategy. Financial Times.

Horton, R., 2020. Coronavirus is the greatest global science policy failure in a generation | Richard Horton. The Guardian.

Hughes, L., 2020. Earlier lockdown could have halved UK deaths, says PM's ex-adviser. Financial Times.

Kaminska, I., 2020. Making sense of nonsensical Covid-19 strategy. Financial Times.

Kelly, J., 2020a. That Imperial coronavirus report, in detail. Financial Times.

Kelly, J., 2020b. Spiegelhalter says majority of Covid deaths would not have occurred in coming year. Financial Times.

Kelly, J., 2020c. Is the "science" behind the lockdown any good? Financial Times.

Kelly, J., 2020d. Is it acceptable for government to be dishonest at a time like this? Financial Times.

Kelly, J., 2020e. 'Following the science' is more complicated than we like to admit. Financial Times.

Kelly, J., Burn-Murdoch, J., 2020. Why are we not wearing masks in the UK? Financial Times.

Kelly, J., Cookson, C., 2020. 'Politicised nature' of lockdown debate delays Imperial report | Free to read. Financial Times.

Landler, M., Castle, S., 2020a. The Secretive Group Guiding the U.K. on Coronavirus. The New York Times.

Landler, M., Castle, S., 2020b. Britain Placed Under a Virtual Lockdown by Boris Johnson. The New York Times.

Landler, M., Castle, S., 2020c. Behind the Virus Report That Jarred the U.S. and the U.K. to Action. The New York Times.

Lawrence, F., Carrell, S., Pegg, D., 2020. Attendees of Sage meetings worried by presence of Cummings. The Guardian.
Lewis, P., Pegg, D., 2020. Google executive took part in Sage meeting, tech firm confirms. The Guardian.
Mason, R., 2020. Government names dozens of scientists who sit on Sage group. The Guardian.
Mueller, B., 2020. As Europe Shuts Down, Britain Takes a Different, and Contentious, Approach. The New York Times.
Neate, R., 2021. Patrick Vallance: the adviser who spoke scientific truth to power. The Guardian.
Parker, G., Bounds, A., Tighe, C., 2020a. Johnson gambles by splitting from his scientists. Financial Times.
Parker, G., Cameron-Chileshe, J., Pickard, J., 2020b. Johnson weighs tougher measures after England lockdown failures. Financial Times.
Parker, G., Neville, S., Powley, T., 2020c. Johnson suffers ‘back to work’ blow from chief scientist. Financial Times.
Parker, G., Payne, S., Pickard, J., 2021. Johnson bows to the inevitable after surge in Covid cases. Financial Times.
Parker, G., Pooler, M., 2020. Gove accused of confusing public with ‘back to work’ message. Financial Times.
Parveen, N., 2020. UK scientists warn of third wave of Covid after Christmas. The Guardian.
Payne, S., Cookson, C., 2020. Boris Johnson shook hands ‘continuously’ despite science panel warnings. Financial Times.
Payne, S., Parker, G., Dickie, M., 2021. Boris Johnson orders third lockdown for England. Financial Times.
Sample, I., 2020a. Who’s who on secret scientific group advising UK government? The Guardian.
Sample, I., 2020b. Government rushes out request for experts to work with Sage panel. The Guardian.
Sample, I., Perraudin, F., Walker, P., Bosely, S., Devlin, H., 2020. Sage minutes reveal how UK advisers reacted to coronavirus crisis. The Guardian.
Sharma, R., 2021. The short shelf life of pandemic national success stories. Financial Times.
Stewart, H., Busby, M., 2020. Coronavirus: science chief defends UK plan from criticism. The Guardian.
Stewart, H., Sample, I., 2020. How No 10’s relationship with its scientists broke down. The Guardian.
Summers, H., 2021. UK government ‘failed to consider gender’ in its response to Covid pandemic. The Guardian.

Supplementary Information 3: SAGE Self-References

The following pages include SAGE's self-references frequencies and keyword comparison

Original data source: SAGE Meeting Minutes (1-89) from UK Government (2021) Scientific evidence supporting the government response to coronavirus (COVID-19). https://www.gov.uk/government/collections/scientific-evidence-supporting-the-government-response-to-coronavirus-covid-19.

SAGE self-reference frequencies

Number of SAGE self-references in each meeting.

TF	FILEID (Meeting No.)	WORD COUNT	SAGE refs freq	NormalisedFreq
1	1	900	5	5.556
1	2	885	10	11.299
1	3	736	1	1.359
1	4	1765	12	6.799
1	5	385	7	18.182
1	6	1552	6	3.866
1	7	1473	8	5.431
1	8	1147	5	4.359
1	9	1163	6	5.159
1	10	1070	6	5.607
1	11	952	5	5.252
1	12	824	5	6.068
1	13	659	8	12.140
1	14	1803	11	6.101
1	15	1864	17	9.120
2	16	1531	7	4.572
2	17	1165	16	13.734
2	18	1686	3	1.779
2	19	1572	8	5.089
2	20	574	4	6.969
2	21	1040	7	6.731
2	22	1300	12	9.231
2	23	1284	11	8.567
2	24	1748	11	6.293
2	25	1618	6	3.708
2	26	1639	15	9.152
2	27	1622	6	3.699
2	28	1264	11	8.703
2	29	1701	16	9.406
2	30	1437	19	13.222
2	31	668	4	5.988
2	32	1196	8	6.689
3	33	1720	8	4.651
3	34	1761	16	9.086
3	35	1999	8	4.002
3	36	1736	19	10.945
3	37	1142	8	7.005
3	38	2715	27	9.945
3	39	1941	25	12.880
3	40	2281	15	6.576
3	41	1824	10	5.482
3	42	1904	25	13.130
3	43	1001	3	2.997
3	44	172	2	11.628

TF	FILEID (Meeting No.)	WORD COUNT	SAGE refs freq	NormalisedFreq
3	45	1799	14	7.782
3	46	1954	15	7.677
3	47	1600	8	5.000
3	48	2064	18	8.721
3	49	1559	11	7.056
3	50	1664	10	6.010
3	51	1020	7	6.863
3	52	1724	20	11.601
3	53	1808	8	4.425
3	54	777	3	3.861
3	55	1300	6	4.615
3	56	1956	8	4.090
3	57	2630	13	4.943
4	58	790	3	3.797
4	59	2583	12	4.646
4	60	1977	10	5.058
4	61	1404	7	4.986
4	62	1804	8	4.435
4	63	2871	11	3.831
4	64	2387	6	2.514
4	65	1027	6	5.842
4	66	2566	10	3.897
4	67	3206	11	3.431
4	68	1006	4	3.976
4	69	3461	11	3.178
4	70	2922	17	5.818
4	71	1802	5	2.775
4	72	2762	7	2.534
4	73	3478	2	0.575
5	74	936	0	0.000
5	75	2105	2	0.950
5	76	3037	6	1.976
5	77	2391	1	0.418
5	78	2872	9	3.134
5	79	3580	5	1.397
5	80	4251	10	2.352
5	81	1720	0	0.000
5	82	1129	2	1.771
5	83	2633	1	0.380
5	84	3030	3	0.990
5	85	1419	0	0.000
5	86	2429	5	2.058
5	87	2851	2	0.702
5	88	1986	4	2.014
5	89	970	1	1.031
**TF**	**TOTAL**	155659	764	

SAGE self-references keyword comparison

Keywords in TF1-3 compared to TF4-5

TF1-3				
**rank**	**freq**	**keyness**	**effect dice**	**Word**
1	132	40.34	0.0164	agreed
2	36	18.64	0.0045	but

TF4-5				
**rank**	**freq**	**keyness**	**effect dice**	**Word**
1	65	84.62	0.0271	has
2	30	80.56	0.0127	see
3	61	79.33	0.0254	previously
4	13	27.73	0.0055	period
5	14	27.15	0.0059	sars
6	19	25.73	0.008	prevalence
7	10	23.41	0.0042	ethnic
8	47	21.58	0.0195	transmission
9	25	19.44	0.0105	considered
10	6	17.75	0.0025	certification
11	6	17.75	0.0025	medium
12	6	17.75	0.0025	minority
13	19	17.33	0.008	children

Supplementary Information 4: Marker Frequency Data

The following pages record the frequency counts for the linguistic markers.

Original data source: SAGE Meeting Minutes (1-89) from UK Government (2021) Scientific evidence supporting the government response to coronavirus (COVID-19). https://www.gov.uk/government/collections/scientific-evidence-supporting-the-government-response-to-coronavirus-covid-19.

Methodology extract from paper: *"*We built on Shen and Tao (2021) to generate an initial list of stance markers and manually inspected the use of each marker. We also assessed whether each marker performed a hedging, boosting or attitudinal function in the context of our data, and added any additional markers specific to our corpus—this includes the explicit mention of uncertainty through confidence intervals labels (e.g., "*low confidence*") introduced in the data partway through our study period. For markers observed to perform multiple functions (e.g., epistemic modals such as "*will*" that can be used as a booster or to talk about possible future scenarios) two researchers checked and classified each instance. The final counts only include those instances in which both annotators agreed functioned exclusively as either a hedging, booster, or attitude marker.*"*

Total frequencies of markers per meeting

TF	FILENAME	COUNT	FILEID	Hedges	Boosters	Attitude	Self-reference	ALL markers	Boosters (norm)	Hedges (norm)	Attitude (norm)	Self-reference (norm)	All markers (norm)
1	1.txt	900	1	27	9	16	5	57	10.000	30.000	17.778	5.556	63.33333333
1	2.txt	885	2	26	3	20	10	59	3.390	29.379	22.599	11.299	66.66666667
1	3.txt	736	3	32	13	25	1	71	17.663	43.478	33.967	1.359	96.4673913
1	4.txt	1765	4	64	20	9	12	105	11.331	36.261	5.099	6.799	59.49008499
1	5.txt	385	5	4	0	11	7	22	0.000	10.390	28.571	18.182	57.14285714
1	6.txt	1552	6	47	20	7	6	80	12.887	30.284	4.510	3.866	51.54639175
1	7.txt	1473	7	36	33	10	8	87	22.403	24.440	6.789	5.431	59.06313646
1	8.txt	1147	8	26	18	7	5	56	15.693	22.668	6.103	4.359	48.82301656
1	9.txt	1163	9	29	20	10	6	65	17.197	24.936	8.598	5.159	55.88993981
1	10.txt	1070	10	26	20	18	6	70	18.692	24.299	16.822	5.607	65.42056075
1	11.txt	952	11	24	12	7	5	48	12.605	25.210	7.353	5.252	50.42016807
1	12.txt	824	12	16	15	7	5	43	18.204	19.417	8.495	6.068	52.18446602
1	13.txt	659	13	9	14	15	8	46	21.244	13.657	22.762	12.14	69.80273141
1	14.txt	1803	14	42	18	5	11	76	9.983	23.295	2.773	6.101	42.15196894
1	15.txt	1864	15	45	17	3	17	82	9.120	24.142	1.609	9.12	43.99141631
2	16.txt	1531	16	33	12	9	7	61	7.838	21.555	5.879	4.572	39.84323971
2	17.txt	1165	17	35	24	18	16	93	20.601	30.043	15.451	13.734	79.82832618
2	18.txt	1686	18	38	19	16	3	76	11.269	22.539	9.490	1.779	45.07710558
2	19.txt	1572	19	24	12	15	8	59	7.634	15.267	9.542	5.089	37.53180662
2	20.txt	574	20	11	8	13	4	36	13.937	19.164	22.648	6.969	62.71777003
2	21.txt	1040	21	13	12	10	7	42	11.538	12.500	9.615	6.731	40.38461538
2	22.txt	1300	22	32	9	8	12	61	6.923	24.615	6.154	9.231	46.92307692
2	23.txt	1284	23	28	10	25	11	74	7.788	21.807	19.470	8.567	57.63239875
2	24.txt	1748	24	38	8	9	11	66	4.577	21.739	5.149	6.293	37.75743707
2	25.txt	1618	25	46	27	11	6	90	16.687	28.430	6.799	3.708	55.62422744
2	26.txt	1639	26	20	16	25	15	76	9.762	12.203	15.253	9.152	46.36973764
2	27.txt	1622	27	40	23	17	6	86	14.180	24.661	10.481	3.699	53.02096178
2	28.txt	1264	28	17	7	7	11	42	5.538	13.449	5.538	8.703	33.2278481
2	29.txt	1701	29	46	9	3	16	74	5.291	27.043	1.764	9.406	43.50382128
2	30.txt	1437	30	37	13	3	19	72	9.047	25.748	2.088	13.222	50.10438413
2	31.txt	668	31	30	4	16	4	54	5.988	44.910	23.952	5.988	80.83832335
2	32.txt	1196	32	24	16	11	8	59	13.378	20.067	9.197	6.689	49.33110368
3	33.txt	1720	33	55	16	14	8	93	9.302	31.977	8.140	4.651	54.06976744
3	34.txt	1761	34	25	30	15	16	86	17.036	14.196	8.518	9.086	48.8358887
3	35.txt	1999	35	48	19	20	8	95	9.505	24.012	10.005	4.002	47.52376188
3	36.txt	1736	36	36	25	14	19	94	14.401	20.737	8.065	10.945	54.14746544
3	37.txt	1142	37	23	7	8	8	46	6.130	20.140	7.005	7.005	40.28021016
3	38.txt	2715	38	54	30	12	27	123	11.050	19.890	4.420	9.945	45.3038674
3	39.txt	1941	39	19	16	23	25	83	8.243	9.789	11.850	12.88	42.76146316
3	40.txt	2281	40	48	25	6	15	94	10.960	21.043	2.630	6.576	41.20999562
3	41.txt	1824	41	43	15	13	10	81	8.224	23.575	7.127	5.482	44.40789474
3	42.txt	1904	42	38	17	17	25	97	8.929	19.958	8.929	13.13	50.94537815
3	43.txt	1001	43	25	21	25	3	74	20.979	24.975	24.975	2.997	73.92607393
3	44.txt	172	44	6	4	4	2	16	23.256	34.884	23.256	11.628	93.02325581
3	45.txt	1799	45	51	19	16	14	100	10.561	28.349	8.894	7.782	55.58643691
3	46.txt	1954	46	39	21	18	15	93	10.747	19.959	9.212	7.677	47.59467758
3	47.txt	1600	47	44	22	17	8	91	13.750	27.500	10.625	5	56.875
3	48.txt	2064	48	56	24	16	18	114	11.628	27.132	7.752	8.721	55.23255814

TF	FILENAME	COUNT	FILEID	Hedges	Boosters	Attitude	Self-reference	ALL markers	Boosters (norm)	Hedges (norm)	Attitude (norm)	Self-reference (norm)	All markers (norm)
3	49.txt	1559	49	46	32	8	11	97	20.526	29.506	5.131	7.056	62.21937139
3	50.txt	1664	50	50	26	19	10	105	15.625	30.048	11.418	6.01	63.10096154
3	51.txt	1020	51	34	12	10	7	63	11.765	33.333	9.804	6.863	61.76470588
3	52.txt	1724	52	46	24	16	20	106	13.921	26.682	9.281	11.601	61.48491879
3	53.txt	1808	53	52	20	25	8	105	11.062	28.761	13.827	4.425	58.07522124
3	54.txt	777	54	15	13	15	3	46	16.731	19.305	19.305	3.861	59.2020592
3	55.txt	1300	55	58	17	12	6	93	13.077	44.615	9.231	4.615	71.53846154
3	56.txt	1956	56	59	35	13	8	115	17.894	30.164	6.646	4.09	58.79345603
3	57.txt	2630	57	91	71	24	13	199	26.996	34.601	9.125	4.943	75.66539924
4	58.txt	790	58	21	17	8	3	49	21.519	26.582	10.127	3.797	62.02531646
4	59.txt	2583	59	75	32	13	12	132	12.389	29.036	5.033	4.646	51.10336818
4	60.txt	1977	60	61	33	20	10	124	16.692	30.855	10.116	5.058	62.72129489
4	61.txt	1404	61	44	29	16	7	96	20.655	31.339	11.396	4.986	68.37606838
4	62.txt	1804	62	72	25	2	8	107	13.858	39.911	1.109	4.435	59.31263858
4	63.txt	2871	63	118	33	20	11	182	11.494	41.101	6.966	3.831	63.39254615
4	64.txt	2387	64	90	38	19	6	153	15.920	37.704	7.960	2.514	64.09719313
4	65.txt	1027	65	20	25	7	6	58	24.343	19.474	6.816	5.842	56.4751704
4	66.txt	2566	66	79	44	9	10	142	17.147	30.787	3.507	3.897	55.3390491
4	67.txt	3206	67	129	34	11	11	185	10.605	40.237	3.431	3.431	57.70430443
4	68.txt	1006	68	33	15	7	4	59	14.911	32.803	6.958	3.976	58.64811133
4	69.txt	3461	69	119	48	21	11	199	13.869	34.383	6.068	3.178	57.497833
4	70.txt	2922	70	99	33	23	17	172	11.294	33.881	7.871	5.818	58.86379192
4	71.txt	1802	71	77	21	0	5	103	11.654	42.730	0.000	2.775	57.15871254
4	72.txt	2762	72	107	51	12	7	177	18.465	38.740	4.345	2.534	64.0839971
4	73.txt	3478	73	100	58	15	2	175	16.676	28.752	4.313	0.575	50.31627372
5	74.txt	936	74	45	11	16	0	72	11.752	48.077	17.094	0	76.92307692
5	75.txt	2105	75	65	23	14	2	104	10.926	30.879	6.651	0.95	49.40617577
5	76.txt	3037	76	90	47	7	6	150	15.476	29.635	2.305	1.976	49.39084623
5	77.txt	2391	77	63	31	21	1	116	12.965	26.349	8.783	0.418	48.51526558
5	78.txt	2872	78	90	45	17	9	161	15.669	31.337	5.919	3.134	56.05849582
5	79.txt	3580	79	97	58	21	5	181	16.201	27.095	5.866	1.397	50.55865922
5	80.txt	4251	80	119	89	31	10	249	20.936	27.993	7.292	2.352	58.57445307
5	81.txt	1720	81	56	34	18	0	108	19.767	32.558	10.465	0	62.79069767
5	82.txt	1129	82	37	10	12	2	61	8.857	32.772	10.629	1.771	54.03011515
5	83.txt	2633	83	92	44	17	1	154	16.711	34.941	6.457	0.38	58.48841626
5	84.txt	3030	84	110	83	13	3	209	27.393	36.304	4.290	0.99	68.97689769
5	85.txt	1419	85	49	31	12	0	92	21.846	34.531	8.457	0	64.83439042
5	86.txt	2429	86	85	33	16	5	139	13.586	34.994	6.587	2.058	57.22519555
5	87.txt	2851	87	99	44	20	2	165	15.433	34.725	7.015	0.702	57.87443002
5	88.txt	1986	88	58	43	13	4	118	21.652	29.204	6.546	2.014	59.41591138
5	89.txt	970	89	35	14	16	1	66	14.433	36.082	16.495	1.031	68.04123711

Boosters' frequency Overview

Booster	Count	Booster	Count	Booster	Count
will	363	extremely	7	finds	1
high_confidence	225	great	7	thinks	1
would	223	demonstrates	6	thought	1
particularly	150	highlights	6	indeed	1
support	138	crucial	6	decisive	1
show	101	demonstrate	5	noteworthy	1
clear	95	find	5	powerful	1
highly	82	completely	5	securely	1
shows	51	definitive	5	thorough	1
certain	51	entirely	5	the_fact_that	1
fully	46	inevitable	5	concludes_that	1
confirmed	44	safely	5	in_fact	1
essential	42	impossible	4	establishes	0
known	33	markedly	4	proved	0
confirm	33	notably	4	believed	0
especially	24	unavoidable	4	knew	0
establish	23	demonstrated	3	realise	0
reliable	23	showed	3	realised	0
considerable	20	think	3	realises	0
total	20	absolutely	3	confirms	0
credible	19	never	3	corroborates	0
meaningful	18	truly	3	corroborated	0
secure	17	entire	3	corroborate	0
highlighted	15	precisely	3	deserve	0
clearly	15	unique	3	manifests	0
enhanced	15	prove	2	manifested	0
supports	14	believe	2	manifest	0
true.	14	always	2	proof	0
safe	12	considerably	2	uphold	0
found	11	obvious	2	upheld	0
shown	11	exceptionally	2	undoubtedly	0
established	10	extensively	2	categorically	0
know	10	firm	2	compelling	0
consistently	9	greatly	2	comprehensively	0
necessarily	9	inevitably	2	conspicuous	0
reliably	9	meaningfully	2	conspicuously	0
highlight	8	outstanding	2	constantly	0
certainly	8	unambiguous	2	convincing	0
comprehensive	8	confidently	2	convincingly	0
marked	8	proves	1	obviously	0
concluded_that	8	believes	1	actually	0

Continues on next page….

Booster	Count	Booster	Count	Booster	Count
conclusively	0	manifestly	0	no_doubt	0
decidedly	0	notable	0	of_course	0
definite	0	noticeable	0	without_doubt	0
definitely	0	noticeably	0	beyond_doubt	0
definitively	0	perfectly	0		
doubtless	0	persuasively	0		
doubtlessly	0	plainly	0		
evident	0	profound	0		
evidently	0	profoundly	0		
incontestable	0	prominent	0		
incontestably	0	prominently	0		
indisputable	0	radical	0		
indisputably	0	radically	0		
really	0	remarkable	0		
surely	0	remarkably	0		
undeniable	0	rigorous	0		
undeniably	0	rigorously	0		
undisputedly	0	self-evident	0		
undoubtedly	0	sizable	0		
credibly	0	sizably	0		
crucially	0	superior	0		
decisively	0	thoroughly	0		
deservedly	0	totally	0		
distinctively	0	unambiguously	0		
essentially	0	unarguably	0		
exhaustively	0	unavoidably	0		
extraordinary	0	undeniable	0		
firmly	0	undeniably	0		
forceful	0	unequivocal	0		
forcefully	0	unequivocally	0		
fundamentally	0	uniquely	0		
genuine	0	unlimited	0		
genuinely	0	unlimitedly	0		
impossibly	0	unmistakable	0		
impressive	0	unmistakably	0		
impressively	0	unprecedented	0		
incontrovertible	0	unquestionable	0		
incontrovertibly	0	unquestionably	0		
indispensable	0	vastly	0		
indispensably	0	vitally	0		
manifest	0	conclude_that	0		

Hedges’ frequency overview

Hedge	Count	Hedge	Count	Hedge	Count
may	500	probably	17	typical	3
likely	387	assumed	16	in_most_cases	3
some	361	dependent_on	16	difficult_to_assess	3
estimates	280	not_possible	15	appeared	2
could	277	plausible	12	claim	2
can	250	largely	12	presumed	2
around	223	sufficiently	12	apparently	2
possible	152	is_not_yet_known	12	normally	2
estimate	132	apparent	11	difficult_to_estimate	2
estimate	132	typically	11	on_balance	2
overall	113	broadly	11	argue	1
suggests	107	approximately	11	argues	1
suggest	92	assumption	9	predicated	1
uncertainty	87	usual	9	inconclusive	1
might	76	is_not_known	9	fairly	1
about	75	seems	8	difficult_to_ascertain	1
estimated	60	probability	8	hard_to_identify	1
expected	58	hard_to_interpret	8	argued	0
rather	57	not_clear	8	claimed	0
low_confidence	51	predict	7	claims	0
indicate	48	generally	7	conjecture	0
indicates	48	not_yet_clear	6	conjectured	0
unlikely	43	indicated	5	conjectures	0
sufficient	43	roughly	5	deduce	0
likelihood	41	usually	5	deduced	0
sensitivity	38	assume	4	doubt	0
depends_on	38	expect	4	doubted	0
relatively	33	inferred	4	doubts	0
often	31	seem	4	guess	0
appears	30	maybe	4	hypothesize	0
general	28	sensitivities	4	hypothesized	0
appear	27	mostly	4	implied	0
unclear	27	mainly	4	implies	0
unknown	27	in_general	4	infers	0
almost	24	difficult_to_quantify	4	postulate	0
indication	23	not_yet_possible	4	postulated	0
possibility	22	assumes	3	postulates	0
uncertain	22	imply	3	predicates	0
uncertain	22	infer	3	presume	0
suggested	18	probable	3	presumes	0
possibly	18	quite	3	reckon	0

Continues on next page….

Hedge	Count	Hedge	Count
reckoned	0	still_needs_to_be_studied	0
speculate	0	open_to_question	0
speculated	0	in_our_view	0
speculates	0	in_my_view	0
suppose	0	in_my_opinion	0
supposed	0	from_this_perspective	0
supposes	0	from_our_perspective	0
surmise	0	from_my_perspective	0
surmised	0		
suspect	0		
suspects	0		
tend to	0		
tended to	0		
tends to	0		
perhaps	0		
essentially	0		
seemingly	0		
presumably	0		
arguably	0		
doubtful	0		
questionable	0		
unclearly	0		
uncertainly	0		
presumable	0		
plausibly	0		
hypothetically	0		
conceivably	0		
essentially	0		
somewhat	0		
sometimes	0		
ocassional	0		
occasionally	0		
certain_amount	0		
on_the_whole	0		
certain_extent	0		
certain_level	0		
certaint_degree	0		
in_most_instances	0		
in_this_view	0		
to_my_knowledge	0		
to_our_knowledge	0		

Attitude markers' frequency overview

Att. Marker	Count	Att. Marker	Count
should	458	unusually	0
important	238	striking	0
will (finalised)	168	strikingly	0
sage_agreed	118	amazed	0
would (finalised)	72	amazing	0
appropriate	65	astonishing	0
expected	58	astonished	0
must	16	curious	0
have_to	9	disappointed	0
correctly	5	disappionting	0
desirable	5	dramatic	0
preferable	5	fortunate	0
appropriately	4	hopeful	0
it_agreed	4	inappropriate	0
successfully	3	interesting	0
understandable	2	remarkable	0
importantly	1	shocking	0
unusual	1	shocked	0
sage_agree	1	striking	0
ought_to	0	surprising	0
admittedly	0	surprised	0
amazingly	0	unbelievable	0
astonishingly	0	unexpected	0
curiously	0	unfortunate	0
desirably	0		
disappointingly	0		
dramatically	0		
expectedly	0		
fortunately	0		
hopefully	0		
inappropriately	0		
interestingly	0		
preferably	0		
remarkably	0		
shockingly	0		
strikingly	0		
surprisingly	0		
unbelievably	0		
understandably	0		
unexpectedly	0		
unfortunately	0		

Supplementary Information 5: SAGE meeting minutes - Time of release

The following pages record the date of SAGE's meeting, URL to the minutes and the release date.

Meeting number	meeting-date	minute-url	publication-date	delay (days)
1	22/01/2020	https://www.gov.uk/government/publications/precautionary-sage-minutes-coronavirus-covid-19-response-22-january-2020/precautionary-sage-1-minutes-coronavirus-covid-19-response-22-january-2020	29/05/2020	128
2	28/01/2020	https://www.gov.uk/government/publications/sage-minutes-coronavirus-covid-19-response-28-january-2020/sage-2-minutes-coronavirus-covid-19-response-28-january-2020	29/05/2020	122
3	03/02/2020	https://www.gov.uk/government/publications/sage-minutes-coronavirus-covid-19-response-3-february-2020/sage-3-minutes-coronavirus-covid-19-response-3-february-2020	29/05/2020	116
4	04/02/2020	https://www.gov.uk/government/publications/sage-minutes-coronavirus-covid-19-response-4-february-2020/sage-4-minutes-coronavirus-covid-19-response-4-february-2020	29/05/2020	115
5	06/02/2020	https://www.gov.uk/government/publications/sage-minutes-coronavirus-covid-19-response-6-february-2020/sage-5-minutes-coronavirus-covid-19-response-6-february-2020	29/05/2020	113
6	11/02/2020	https://www.gov.uk/government/publications/sage-minutes-coronavirus-covid-19-response-11-february-2020/sage-6-minutes-coronavirus-covid-19-response-11-february-2020	29/05/2020	108
7	13/02/2020	https://www.gov.uk/government/publications/sage-minutes-coronavirus-covid-19-response-13-february-2020/sage-7-minutes-coronavirus-covid-19-response-13-february-2020	29/05/2020	106
8	18/02/2020	https://www.gov.uk/government/publications/sage-minutes-coronavirus-covid-19-response-18-february-2020/sage-8-minutes-coronavirus-covid-19-response-18-february-2020	29/05/2020	101
9	20/02/2020	https://www.gov.uk/government/publications/sage-minutes-coronavirus-covid-19-response-20-february-2020/sage-9-minutes-coronavirus-covid-19-response-20-february-2020	29/05/2020	99
10	25/02/2020	https://www.gov.uk/government/publications/sage-minutes-coronavirus-covid-19-response-25-february-2020/sage-10-minutes-coronavirus-covid-19-response-25-february-2020	29/05/2020	94
11	27/02/2020	https://www.gov.uk/government/publications/sage-minutes-coronavirus-covid-19-response-27-february-2020/sage-11-minutes-coronavirus-covid-19-response-27-february-2020	29/05/2020	92
12	03/03/2020	https://www.gov.uk/government/publications/sage-minutes-coronavirus-covid-19-response-3-march-2020/sage-12-minutes-coronavirus-covid-19-response-3-march-2020	29/05/2020	87
13	05/03/2020	https://www.gov.uk/government/publications/sage-minutes-coronavirus-covid-19-5-march-2020/sage-13-minutes-coronavirus-covid-19-response-5-march-2020	29/05/2020	85
14	10/03/2020	https://www.gov.uk/government/publications/sage-minutes-coronavirus-covid-19-response-10-march-2020/sage-14-minutes-coronavirus-covid-19-response-10-march-2020	29/05/2020	80

Meeting number	meeting-date	minute-url	publication-date	delay (days)
15	13/03/2020	https://www.gov.uk/government/publications/sage-minutes-coronavirus-covid-19-response-13-march-2020/sage-15-minutes-coronavirus-covid-19-response-13-march-2020	29/05/2020	77
16	16/03/2020	https://www.gov.uk/government/publications/sage-minutes-coronavirus-covid-19-response-16-march-2020/sage-16-minutes-coronavirus-covid-19-response-16-march-2020	29/05/2020	74
17	18/03/2020	https://www.gov.uk/government/publications/sage-minutes-coronavirus-covid-19-response-18-march-2020/sage-17-minutes-coronavirus-covid-19-response-18-march-2020	29/05/2020	72
18	23/03/2020	https://www.gov.uk/government/publications/sage-minutes-coronavirus-covid-19-response-23-march-2020/sage-18-minutes-coronavirus-covid-19-response-23-march-2020	29/05/2020	67
19	26/03/2020	https://www.gov.uk/government/publications/sage-minutes-coronavirus-covid-19-response-26-march-2020/sage-19-minutes-coronavirus-covid-19-response-26-march-2020	29/05/2020	64
20	29/03/2020	https://www.gov.uk/government/publications/sage-minutes-coronavirus-covid-19-response-29-march-2020/sage-20-minutes-coronavirus-covid-19-response-29-march-2020	29/05/2020	61
21	31/03/2020	https://www.gov.uk/government/publications/sage-minutes-coronavirus-covid-19-response-31-march-2020/sage-21-minutes-coronavirus-covid-19-response-31-march-2020	29/05/2020	59
22	02/04/2020	https://www.gov.uk/government/publications/sage-minutes-coronavirus-covid-19-response-2-april-2020/sage-22-minutes-coronavirus-covid-19-response-2-april-2020	29/05/2020	57
23	07/04/2020	https://www.gov.uk/government/publications/sage-minutes-coronavirus-covid-19-response-7-april-2020/sage-23-minutes-coronavirus-covid-19-response-7-april-2020	29/05/2020	52
24	09/04/2020	https://www.gov.uk/government/publications/sage-minutes-coronavirus-covid-19-response-9-april-2020/sage-24-minutes-coronavirus-covid-19-response-9-april-2020	29/05/2020	50
25	14/04/2020	https://www.gov.uk/government/publications/sage-minutes-coronavirus-covid-19-response-14-april-2020/sage-25-minutes-coronavirus-covid-19-response-14-april-2020	29/05/2020	45
26	16/04/2020	https://www.gov.uk/government/publications/sage-minutes-coronavirus-covid-19-response-16-april-2020/sage-26-minutes-coronavirus-covid-19-response-16-april-2020	29/05/2020	43
27	21/04/2020	https://www.gov.uk/government/publications/sage-minutes-coronavirus-covid-19-response-21-april-2020/sage-27-minutes-coronavirus-covid-19-response-21-april-2020	29/05/2020	38
28	23/04/2020	https://www.gov.uk/government/publications/sage-minutes-coronavirus-covid-19-response-23-april-2020/sage-28-minutes-coronavirus-covid-19-response-23-april-2020	29/05/2020	36
29	28/04/2020	https://www.gov.uk/government/publications/sage-minutes-coronavirus-covid-19-response-28-april-2020/sage-29-minutes-coronavirus-covid-19-response-28-april-2020	29/05/2020	31
30	30/04/2020	https://www.gov.uk/government/publications/sage-minutes-coronavirus-covid-19-response-30-april-2020/sage-30-minutes-coronavirus-covid-19-response-30-april-2020	29/05/2020	29
31	01/05/2020	https://www.gov.uk/government/publications/sage-minutes-coronavirus-covid-19-response-1-may-2020/sage-31-minutes-coronavirus-covid-19-response-1-may-2020	29/05/2020	28
32	01/05/2020	https://www.gov.uk/government/publications/sage-minutes-coronavirus-covid-19-response-1-may-2020--2/sage-32-minutes-coronavirus-covid-19-response-1-may-2020	29/05/2020	28

Meeting number	meeting-date	minute-url	publication-date	delay (days)
33	05/05/2020	https://www.gov.uk/government/publications/sage-minutes-coronavirus-covid-19-response-5-may-2020/sage-33-minutes-coronavirus-covid-19-response-5-may-2020	29/05/2020	24
34	07/05/2020	https://www.gov.uk/government/publications/sage-minutes-coronavirus-covid-19-response-7-may-2020/sage-34-minutes-coronavirus-covid-19-response-7-may-2020	29/05/2020	22
35	12/05/2020	https://www.gov.uk/government/publications/sage-35-minutes-coronavirus-covid-19-response-12-may-2020/sage-35-minutes-coronavirus-covid-19-response-12-may-2020	12/06/2020	31
36	14/05/2020	https://www.gov.uk/government/publications/sage-36-minutes-coronavirus-covid-19-response-14-may-2020/sage-36-minutes-coronavirus-covid-19-response-14-may-2020	12/06/2020	29
37	19/05/2020	https://www.gov.uk/government/publications/sage-37-minutes-coronavirus-covid-19-response-19-may-2020/sage-37-minutes-coronavirus-covid-19-response-19-may-2020	19/06/2020	31
38	21/05/2020	https://www.gov.uk/government/publications/sage-38-minutes-coronavirus-covid-19-response-21-may-2020/sage-38-minutes-coronavirus-covid-19-response-21-may-2020	19/06/2020	29
39	28/05/2020	https://www.gov.uk/government/publications/sage-39-minutes-coronavirus-covid-19-response-28-may-2020/sage-39-minutes-coronavirus-covid-19-response-28-may-2020	26/06/2020	29
40	04/06/2020	https://www.gov.uk/government/publications/sage-40-minutes-coronavirus-covid-19-response-4-june-2020/sage-40-minutes-coronavirus-covid-19-response-4-june-2020	26/06/2020	22
41	11/06/2020	https://www.gov.uk/government/publications/sage-41-minutes-coronavirus-covid-19-response-11-june-2020/sage-41-minutes-coronavirus-covid-19-response-11-june-2020	26/06/2020	15
42	18/06/2020	https://www.gov.uk/government/publications/sage-42-minutes-coronavirus-covid-19-response-18-june-2020/sage-42-minutes-coronavirus-covid-19-response-18-june-2020	14/08/2020	57
43	23/06/2020	https://www.gov.uk/government/publications/sage-43-minutes-coronavirus-covid-19-response-23-june-2020/sage-43-minutes-coronavirus-covid-19-response-23-june-2020	31/07/2020	38
44	25/06/2020	https://www.gov.uk/government/publications/sage-44-minutes-coronavirus-covid-19-response-25-june-2020/sage-44-minutes-coronavirus-covid-19-response-25-june-2020	31/07/2020	36
45	02/07/2020	https://www.gov.uk/government/publications/sage-45-minutes-coronavirus-covid-19-response-2-july-2020/forty-fifth-sage-meeting-on-covid-19-2-july-2020	31/07/2020	29
46	09/07/2020	https://www.gov.uk/government/publications/sage-46-minutes-coronavirus-covid-19-response-9-july-2020/sage-46-minutes-coronavirus-covid-19-response-9-july-2020	03/08/2020	25
47	16/07/2020	https://www.gov.uk/government/publications/sage-47-minutes-coronavirus-covid-19-response-16-july-2020/sage-47-minutes-coronavirus-covid-19-response-16-july-2020	25/09/2020	71
48	23/07/2020	https://www.gov.uk/government/publications/sage-48-minutes-coronavirus-covid-19-response-23-july-2020/sage-48-minutes-coronavirus-covid-19-response-23-july-2020	25/09/2020	64
49	30/07/2020	https://www.gov.uk/government/publications/sage-49-minutes-coronavirus-covid-19-response-30-july-2020/sage-49-minutes-coronavirus-covid-19-response-30-july-2020	11/12/2020	134
50	06/08/2020	https://www.gov.uk/government/publications/sage-50-minutes-coronavirus-covid-19-response-6-august-2020/sage-50-minutes-coronavirus-covid-19-response-6-august-2020	25/09/2020	50

Meeting number	meeting-date	minute-url	publication-date	delay (days)
51	13/08/2020	https://www.gov.uk/government/publications/sage-51-minutes-coronavirus-covid-19-response-13-august-2020/sage-51-minutes-coronavirus-covid-19-response-13-august-2020	25/09/2020	43
52	20/08/2020	https://www.gov.uk/government/publications/sage-52-minutes-coronavirus-covid-19-response-20-august-2020/sage-52-minutes-coronavirus-covid-19-response-20-august-2020	18/09/2020	29
53	27/08/2020	https://www.gov.uk/government/publications/sage-53-minutes-coronavirus-covid-19-response-27-august-2020/sage-53-minutes-coronavirus-covid-19-response-27-august-2020	18/09/2020	22
54	01/09/2020	https://www.gov.uk/government/publications/sage-54-minutes-coronavirus-covid-19-response-1-september-2020/sage-54-minutes-coronavirus-covid-19-response-1-september-2020	18/09/2020	17
55	03/09/2020	https://www.gov.uk/government/publications/sage-55-minutes-coronavirus-covid-19-response-3-september-2020/sage-55-minutes-coronavirus-covid-19-response-3-september-2020	18/09/2020	15
56	10/09/2020	https://www.gov.uk/government/publications/sage-56-minutes-coronavirus-covid-19-response-10-september-2020/sage-56-minutes-coronavirus-covid-19-response-10-september-2020	23/10/2020	43
57	17/09/2020	https://www.gov.uk/government/publications/sage-57-record-of-meeting-on-covid-19-17-september-2020/sage-57-minutes-coronavirus-covid-19-response-17-september-2020	30/10/2020	43
58	17/09/2020	https://www.gov.uk/government/publications/fifty-eighth-sage-meeting-on-covid-19-21-september-2020/sage-58-minutes-coronavirus-covid-19-response-21-september-2020	12/10/2020	25
59	24/09/2020	https://www.gov.uk/government/publications/sage-59-minutes-coronavirus-covid-19-response-24-september-2020/sage-59-minutes-coronavirus-covid-19-response-24-september-2020	30/10/2020	36
60	01/10/2020	https://www.gov.uk/government/publications/sage-60-minutes-coronavirus-covid-19-response-1-october-2020/sixtieth-sage-meeting-on-covid-19-1-october-2020	30/10/2020	29
61	08/10/2020	https://www.gov.uk/government/publications/sage-61-minutes-coronavirus-covid-19-response-8-october-2020/sixty-first-sage-meeting-on-covid-19-8-october-2020	30/10/2020	22
62	15/10/2020	https://www.gov.uk/government/publications/sage-62-minutes-coronavirus-covid-19-response-15-october-2020/sixty-second-sage-meeting-on-covid-19-15-october-2020	13/11/2020	29
63	22/10/2020	https://www.gov.uk/government/publications/sage-63-minutes-coronavirus-covid-19-response-22-october-2020/sixty-third-sage-meeting-on-covid-19-22-october-2020	13/11/2020	22
64	29/10/2020	https://www.gov.uk/government/publications/sage-64-minutes-coronavirus-covid-19-response-29-october-2020/sixty-fourth-sage-meeting-on-covid-19-29-october-2020	27/11/2020	29
65	04/11/2020	https://www.gov.uk/government/publications/sage-65-minutes-coronavirus-covid-19-response-4-november-2020/sixty-fifth-sage-meeting-on-covid-19-4-november-2020	13/11/2020	9
66	05/11/2020	https://www.gov.uk/government/publications/sage-66-minutes-coronavirus-covid-19-response-5-november-2020/sixty-sixth-sage-meeting-on-covid-19-5-november-2020	27/11/2020	22
67	12/11/2020	https://www.gov.uk/government/publications/sage-67-minutes-coronavirus-covid-19-response-12-november-2020/sixty-seventh-sage-meeting-on-covid-19-12-november-2020	23/11/2020	11
68	16/11/2020	https://www.gov.uk/government/publications/sage-68-minutes-coronavirus-covid-19-response-16-november-2020/sixty-eighth-sage-meeting-on-covid-19-16-november-2020	18/12/2020	32

Meeting number	meeting-date	minute-url	publication-date	delay (days)
69	19/11/2020	https://www.gov.uk/government/publications/sage-69-minutes-coronavirus-covid-19-response-19-november-2020/sixty-ninth-sage-meeting-on-covid-19-19-november-2020	27/11/2020	8
70	26/11/2020	https://www.gov.uk/government/publications/sage-70-minutes-coronavirus-covid-19-response-26-november-2020/sage-70-minutes-coronavirus-covid-19-response-26-november-2020	18/12/2020	22
71	03/12/2020	https://www.gov.uk/government/publications/sage-71-minutes-coronavirus-covid-19-response-3-december-2020/sage-71-minutes-coronavirus-covid-19-response-3-december-2020	18/12/2020	15
72	10/12/2020	https://www.gov.uk/government/publications/sage-72-minutes-coronavirus-covid-19-response-10-december-2020/sage-72-minutes-coronavirus-covid-19-response-10-december-2020	05/07/2021	207
73	17/12/2020	https://www.gov.uk/government/publications/sage-73-minutes-coronavirus-covid-19-response-17-december-2020/sage-73-minutes-coronavirus-covid-19-response-17-december-2020	15/01/2021	29
74	22/12/2020	https://www.gov.uk/government/publications/sage-74-minutes-coronavirus-covid-19-response-22-december-2020/sage-74-minutes-coronavirus-covid-19-response-22-december-2020	31/12/2020	9
75	07/01/2021	https://www.gov.uk/government/publications/sage-75-minutes-coronavirus-covid-19-response-7-january-2021/sage-75-minutes-coronavirus-covid-19-response-7-january-2021	22/01/2021	15
76	14/01/2021	https://www.gov.uk/government/publications/sage-76-minutes-coronavirus-covid-19-response-14-january-2021/sage-76-minutes-coronavirus-covid-19-response-14-january-2021	29/01/2021	15
77	21/01/2021	https://www.gov.uk/government/publications/sage-77-minutes-coronavirus-covid-19-response-21-january-2021/sage-77-minutes-coronavirus-covid-19-response-21-january-2021	05/02/2021	15
78	28/01/2021	https://www.gov.uk/government/publications/sage-78-minutes-coronavirus-covid-19-response-28-january-2021/sage-78-minutes-coronavirus-covid-19-response-28-january-2021	22/02/2021	25
79	04/02/2021	https://www.gov.uk/government/publications/sage-79-minutes-coronavirus-covid-19-response-4-february-2021/sage-79-minutes-coronavirus-covid-19-response-4-february-2021	22/02/2021	18
80	11/02/2021	https://www.gov.uk/government/publications/sage-80-minutes-coronavirus-covid-19-response-11-february-2021/sage-80-minutes-coronavirus-covid-19-response-11-february-2021	26/02/2021	15
81	18/02/2021	https://www.gov.uk/government/publications/sage-81-minutes-coronavirus-covid-19-response-18-february-2021/sage-81-minutes-coronavirus-covid-19-response-18-february-2021	22/02/2021	4
82	25/02/2021	https://www.gov.uk/government/publications/sage-82-minutes-coronavirus-covid-19-response-25-february-2021/sage-82-minutes-coronavirus-covid-19-response-25-february-2021	12/03/2021	15
83	11/03/2021	https://www.gov.uk/government/publications/sage-83-minutes-coronavirus-covid-19-response-11-march-2021/sage-83-minutes-coronavirus-covid-19-response-11-march-2021	26/03/2021	15
84	25/03/2021	https://www.gov.uk/government/publications/sage-84-minutes-coronavirus-covid-19-response-25-march-2021/sage-84-minutes-coronavirus-covid-19-response-25-march-2021	09/04/2021	15
85	31/03/2021	https://www.gov.uk/government/publications/eighty-fifth-sage-meeting-on-covid-19-31-march-2021/eighty-fifth-sage-meeting-on-covid-19-31-march-2021	09/04/2021	9
86	08/04/2021	https://www.gov.uk/government/publications/sage-86-minutes-coronavirus-covid-19-response-8-april-2021/sage-86-minutes-coronavirus-covid-19-response-8-april-2021	23/04/2021	15

Meeting number	meeting-date	minute-url	publication-date	delay (days)
87	22/04/2021	https://www.gov.uk/government/publications/sage-87-minutes-coronavirus-covid-19-response-22-april-2021/sage-87-minutes-coronavirus-covid-19-response-22-april-2021	05/07/2021	74
88	05/05/2021	https://www.gov.uk/government/publications/sage-88-minutes-coronavirus-covid-19-response-5-may-2021/sage-88-minutes-coronavirus-covid-19-response-5-may-2021	10/05/2021	5
89	13/05/2021	https://www.gov.uk/government/publications/sage-89-minutes-coronavirus-covid-19-response-13-may-2021/sage-89-minutes-coronavirus-covid-19-response-13-may-2021	14/05/2021	1

Supplementary Information 6: People attending SAGE meetings

The following pages record the institution categorisations and number of redacted names.

Original data source: SAGE Meeting Minutes (1-89) from UK Government (2021) Scientific evidence supporting the government response to coronavirus (COVID-19). https://www.gov.uk/government/collections/scientific-evidence-supporting-the-government-response-to-coronavirus-covid-19.

Note: The roles of some attendees switched from experts to observers and vice versa during the process – therefore, in our final count, we only accounted for each attendee's most frequent role

Institution Categories

Categorisation (by authors) of expert's institutions listed on SAGE's website (applicable to Fig. 3).

Institution	Type of Institution (assigned by authors)
**Public Health England**	Public Health
**Department of Health and Social Care (DHSC)**	UK Departments
**Scottish Government**	Devolved Nations Government
**London School of Hygiene and Tropical Medicine**	Universities
**Cabinet Office**	UK Government
**GO-Science**	Government Scientific Advisory
**No. 10**	UK Government
**University of Cambridge**	Universities
**Department for Education (DfE)**	UK Departments
**Joint Biosecurity Centre (JBC)**	Government Scientific Advisory
**University of Oxford**	Universities
**Welsh Government**	Devolved Nations Government
**University College London**	Universities
**Foreign, Commonwealth and Development Office (FCDO)**	UK Departments
**HM Treasury**	UK Departments
**Imperial College London**	Universities
**Department for Transport (DfT)**	UK Departments
**National Health Service (NHS)**	Public Health
**Office for National Statistics (ONS)**	Non-ministerial Government Departments
**Home Office**	UK Departments
**University of Edinburgh**	Universities
**University of Liverpool**	Universities
**University of Leeds**	Universities
**Royal Society**	National Academies of Sciences
**NHS Test and Trace**	Public Health
**HDR-UK**	Public Health

**Department for Environment, Food and Rural Affairs (DEFRA)**	UK Departments
**Department for Business, Energy and Industrial Strategy (BEIS)**	UK Departments
**Department for International Development**	UK Departments
**Kings College London**	Universities
**Deputy Chief Medical Officer**	Government Scientific Advisory
**Ministry of Defence**	UK Departments
**University of Manchester**	Universities
**Health and Safety Executive**	Public Health
**NHSx**	Public Health
**Ministry for Housing, Communities and Local Government**	UK Departments
**University of St Andrews**	Universities
**UK Research and Innovation**	Non-departmental Public Body
**University of Birmingham**	Universities
**University of Dundee**	Universities
**University of Glasgow**	Universities
**Northern Ireland Department of Health**	Public Health Devolved Nations
**Public Health Wales**	Public Health Devolved Nations
**Government Chief Scientific Adviser**	Government Scientific Advisory
**NHS**	Public Health
**Chief Veterinary Officer**	Government Scientific Advisory
**Liverpool School of Tropical Medicine**	Universities
**Wellcome Trust**	Foundations
**Health Protection Scotland**	Public Health Devolved Nations
**European Centre for Disease Prevention and Control**	European Institutions
**Civil Contingencies Secretariat**	UK Government
**Food Standards Agency**	Non-ministerial Government Departments
**Scottish Government Chief Medical Officer**	Public Health Devolved Nations
**Behavioural Insights Team**	UK Government
**Department for Digital, Culture, Media and Sport**	UK Departments
**in personal capacity**	Other
**University of Bristol and University of Southampton**	Universities

**Northern Ireland Executive**	Devolved Nations Government
**HM Government**	UK Government
**University of Surrey**	Universities
**UWE Bristol**	Universities
**Deputy Director General EMBL**	Research Institutes
**Keele University**	Universities
**University of Bristol**	Universities
**Met Office**	Government Executive Agency
**University of Southhampton**	Universities
**Birmingham City University**	Universities
**Government Actuary Department**	Non-ministerial Government Departments
**Institute and Faculty of Actuaries**	Professional Bodies
**University of Strathclyde**	Universities
**Association of Directors of Public Health**	Professional Bodies
**University of Leicester**	Universities
**Genomics England**	Government-owned Companies
**University of Bath**	Universities
**University of Nottingham**	Universities
**Northern Ireland Government**	Devolved Nations Government
**University of Warwick**	Universities
**London School of Economics**	Universities
**Queen Mary University of London**	Universities

Redacted name count

The number of redacted names for experts, observers, and secretariat in each meeting.

Meeting number	Experts listed	Observer listed	Secretaries listed	Observers redacted	Secretariat redacted	Experts redacted	Sum listed	Sum redacted	Sum total
**1**	18	5	0	6	5	0	23	11	34
**2**	17	3	0	0	4	0	20	4	24
**3**	9	4	0	0	4	0	13	4	17
**4**	17	5	0	5	6	0	22	11	33
**5**	8	1	0	4	7	0	9	11	20
**6**	14	2	0	0	4	1	16	5	21
**7**	13	1	0	0	3	0	14	3	17
**8**	16	2	0	0	6	3	18	9	27
**9**	16	1	0	0	4	3	17	7	24
**10**	15	1	0	0	5	2	16	7	23
**11**	14	1	0	0	5	3	15	8	23
**12**	18	1	0	1	0	1	19	2	21
**13**	14	4	0	0	6	2	18	8	26
**14**	21	1	0	0	5	3	22	8	30
**15**	23	1	0	0	5	1	24	6	30
**16**	23	2	0	0	5	1	25	6	31
**17**	23	4	0	1	5	1	27	7	34
**18**	24	2	0	1	7	2	26	10	36
**19**	22	2	0	0	0	0	24	0	24
**20**	16	1	0	0	4	0	17	4	21
**21**	24	5	0	0	5	0	29	5	34
**22**	27	2	0	0	4	0	29	4	33
**23**	22	1	0	0	5	0	23	5	28
**24**	23	2	0	0	5	0	25	5	30
**25**	34	3	0	0	6	0	37	6	43
**26**	32	3	0	0	5	0	35	5	40
**27**	36	1	0	0	5	0	37	5	42
**28**	33	1	0	0	5	0	34	5	39
**29**	34	6	0	3	6	0	40	9	49
**30**	38	4	1	4	8	2	43	14	57
**31**	21	6	2	4	9	0	29	13	42
**32**	19	2	1	4	8	1	22	13	35
**33**	31	4	2	3	9	0	37	12	49
**34**	30	3	2	4	10	1	35	15	50
**35**	32	3	3	6	13	0	38	19	57
**36**	32	2	2	4	11	0	36	15	51
**37**	35	2	3	4	11	0	40	15	55
**38**	37	4	2	4	13	0	43	17	60
**39**	37	5	3	7	13	0	45	20	65
**40**	48	5	2	7	14	0	55	21	76
**41**	37	3	2	5	14	1	42	20	62
**42**	35	2	2	6	19	2	39	27	66
**43**	34	2	3	7	15	2	39	24	63
**44**	7	0	0	1	5	0	7	6	13

Meeting number	Experts listed	Observer listed	Secretaries listed	Observers redacted	Secretariat redacted	Experts redacted	Sum listed	Sum redacted	Sum total
**45**	37	1	1	7	13	0	39	20	59
**46**	40	4	2	7	17	1	46	25	71
**47**	29	2	2	7	15	0	33	22	55
**48**	37	1	2	8	14	1	40	23	63
**49**	29	1	3	8	15	1	33	24	57
**50**	27	2	1	8	13	3	30	24	54
**51**	17	3	1	6	13	1	21	20	41
**52**	24	2	2	7	12	0	28	19	47
**53**	30	3	1	7	14	0	34	21	55
**54**	22	3	1	7	11	0	26	18	44
**55**	27	1	1	6	12	0	29	18	47
**56**	33	6	1	8	11	0	40	19	59
**57**	34	5	2	6	12	0	41	18	59
**58**	27	5	2	5	12	0	34	17	51
**59**	33	7	2	10	14	0	42	24	66
**60**	36	9	2	6	12	1	47	19	66
**61**	31	14	2	4	12	0	47	16	63
**62**	32	14	1	7	12	1	47	20	67
**63**	32	11	1	4	14	0	44	18	62
**64**	28	13	2	9	18	0	43	27	70
**65**	32	12	2	7	12	1	46	20	66
**66**	33	7	2	8	15	0	42	23	65
**67**	33	14	3	9	15	1	50	25	75
**68**	28	9	2	6	12	0	39	18	57
**69**	36	15	2	6	17	4	53	27	80
**70**	40	9	2	5	19	0	51	24	75
**71**	36	12	3	10	19	0	51	29	80
**72**	36	12	3	7	15	0	51	22	73
**73**	39	13	3	7	19	1	55	27	82
**74**	31	14	2	7	14	0	47	21	68
**75**	36	14	3	7	19	1	53	27	80
**76**	32	15	2	9	23	1	49	33	82
**77**	35	22	2	8	17	1	59	26	85
**78**	33	17	3	8	19	1	53	28	81
**79**	33	16	2	9	17	1	51	27	78
**80**	39	17	2	11	19	1	58	31	89
**81**	36	19	2	10	15	1	57	26	83
**82**	33	17	1	7	13	1	51	21	72
**83**	41	21	3	8	16	1	65	25	90
**84**	38	18	3	10	19	0	59	29	88
**85**	28	16	2	7	9	1	46	17	63
**86**	37	22	1	9	13	0	60	22	82
**87**	36	20	2	7	16	0	58	23	81
**88**	38	17	3	0	0	0	58	0	58
**89**	39	19	2	14	13	0	60	27	87
